# The Schwann cell-specific G-protein Gαo (Gnao1) is a cell-intrinsic controller contributing to the regulation of myelination in peripheral nerve system

**DOI:** 10.1186/s40478-024-01720-3

**Published:** 2024-02-08

**Authors:** Jinghui Xu, Qianqian Peng, Jieyi Cai, Jianghong Shangguan, Wenfeng Su, Gang Chen, Hualin Sun, Changlai Zhu, Yun Gu

**Affiliations:** Jiangsu Key Laboratory of Neuroregeneration, Co-Innovation Center of Neuroregeneration, Jiangsu Clinical Medicine Center of Tissue Engineering and Nerve Injury Repair, Nantong University, Nantong, JS 226001 People’s Republic of China

**Keywords:** Gnao1, Schwann cells, Differentiation, (Re-)myelination, Cyclic AMP, Demyelinating diseases

## Abstract

Myelin sheath abnormality is the cause of various neurodegenerative diseases (NDDs). G-proteins and their coupled receptors (GPCRs) play the important roles in myelination. Gnao1, encoding the major Gα protein (Gαo) in mammalian nerve system, is required for normal motor function. Here, we show that Gnao1 restricted to Schwann cell (SCs) lineage, but not neurons, negatively regulate SC differentiation, myelination, as well as re-myelination in peripheral nervous system (PNS). Mice lacking Gnao1 expression in SCs exhibit faster re-myelination and motor function recovery after nerve injury. Conversely, mice with Gnao1 overexpression in SCs display the insufficient myelinating capacity and delayed re-myelination. In vitro, Gnao1 deletion in SCs promotes SC differentiation. We found that Gnao1 knockdown in SCs resulting in the elevation of cAMP content and the activation of PI3K/AKT pathway, both associated with SC differentiation. The analysis of RNA sequencing data further evidenced that Gnao1 deletion cause the increased expression of myelin-related molecules and activation of regulatory pathways. Taken together, our data indicate that Gnao1 negatively regulated SC differentiation by reducing cAMP level and inhibiting PI3K-AKT cascade activation, identifying a novel drug target for the treatment of demyelinating diseases.

**Supplementary Information:**

The online version contains supplementary material available at 10.1186/s40478-024-01720-3.

## Introduction

In the central and peripheral nervous system (CNS and PNS), most axons are surrounded by the multilayer of specialized membranes known as the myelin sheath, an organelle produced by Schwann cells (SCs) in PNS and oligodendrocytes (OLs) in CNS, which not only accelerates nerve impulse propagation but is essential for the functional integrity and long term health of axons [33, 43]. Loss or disorder of myelin sheath is the cause of a variety of neurodegenerative diseases (NDDs), including multiple sclerosis (MS), inherited leukodystrophies in the central nerve system, peripheral neuropathies such as Guillain-Barré Syndrome, and other demyelinating diseases caused by the external factors (e.g. trauma, infection or poisoning) and various pathological conditions [35, 52]. Moreover, researchers recently found that even subtle myelin abnormalities may also contribute to more complex neurological disorders, such as schizophrenia and epilepsy [7, 11, 34]. Because of the important role of myelin sheath in neurophysiology, discovering of the mechanisms underlying myelin formation and re-myelination could help to identify new targets for the treatment of neurological disorders. Indeed, enhancing endogenous re-myelination has recently emerged as a promising therapeutic approach in the common but complex NDDs [12, 37, 52].

G-proteins and their coupled receptors (GPCRs), the largest intracellular signal molecule superfamily and by far the most successful drug targets, play the important roles in the development of myelin-forming cells (i.e. SCs and OLs), myelination and re-myelination [21, 23, 26]. For example, GPR126 (also known as ADGRG6) is a conserved regulator of SC myelination in the PNS, which initiates myelination by coupling to the Gα_s_ proteins, increasing cyclic AMP (cAMP) levels and activating protein kinase A (PKA), eventually leading to SC differentiation and myelination [25, 27, 29]. GPR126 mutations in humans cause reduced expression of myelin genes leading to lethal congenital contracture syndrome [41]. In addition, GPR44, activated by prostaglandin D2, play an important role in the formation and maintenance of PNS myelin sheath [47]. In the CNS, GPR56 [1, 13] and GPR17 [6, 49] modulate the proliferation and early differentiation of oligodendrocyte precursor cells (OPCs), and mutations of GPR56 cause bilateral frontoparietal polymicrogyria disease in humans [40]. GPR149 [45] and GPR37 [50] were found to be the negative regulators of OL differentiation and myelination, and genetic deletion of Gpr37 results in precocious oligodendrocyte differentiation and hypermyelination [50]. Undoubtedly, the recent works have highlighted the key roles of GPCRs in nervous system development and demyelinating diseases, but so far, there is a relative paucity of knowledge regarding G-proteins, the couple of GPCRs, in the development of myelin-forming cells and the process of myelination.

G protein complex consists of a Gα subunit and a Gβγ dimer, of which the Gα is responsible for binding to the cognate GPCRs and to guanosine 5′- diphosphate (GDP) or guanosine 5′-triphosphate (GTP). The GDP-GTP exchange on Gα induce the dissociation of Gα-GTP from Gβγ, activates downstream effectors, or restores the Gα to its GDP-bound state, recovers the Gβγ protein, leading to inactivation of downstream signals [3, 17, 36]. Gαo, encoded by gene Gnao1, is the major Gα subunit in the mammalian nerve system, which plays a key role in various physiological functions in health and disease [5, 48]. Mice lacking Gαo (i.e. Gnao1 mutations) exhibit multiple neurological abnormalities, including hyperactivity, generalized tremor, epileptic seizures, severe motor control impairment, and hyperalgesia [4, 48]. In addition, Gnao1 heterozygous mutations are the cause of a severe neurodevelopmental disorder, featuring early infantile seizures and profound cognitive dysfunction [9, 38]. Typically, seizures and movement disorders are the frequent symptoms in demyelinating diseases, such as MS [7], we thus hypothesized that there must be a correlation between Gnao1 and myelination. Currently, little is known about the role of Gnao1 in myelination, so its functions need to be fully elucidated.

Here, we aim to investigate and explain the function of Gnao1 in the PNS myelination, and our data suggest that Gnao1 functions in SCs for timely differentiation and myelination. In a re-myelination model, Gnao1 deficiency in SCs significantly enhances myelin regeneration, but Gnao1 in neurons not. We also show that Gnao1 is a negative regulator of SC differentiation and that its inhibitory function is mediated by reducing cAMP level and suppression of PI3K-AKT cascade activation. To our knowledge, this is the first study reporting the role of Gnao1 in myelination, further suggesting that G proteins and its coupled GPCRs are the important modulators of myelination.

## Materials and methods

### Animals

All experimental animals were provided by the Experimental Animal Center of Nantong University, and subjected to experiments and studies in accordance with the laboratory animal welfare and ethical guidelines approved by the Experimental Animals Administration Committee of Nantong University. The animals were housed in a pathogen-free environments with temperature and humidity controls, ensuring that they have free access to standard chow and water, and in a 12 h/12 h light/dark cycle, monitor their status regularly.

### Adeno-associated virus (AAV) injection

To generate the Gnao1 knockdown AAV, the shRNA targeting Gnao1 (Gnao1-shRNA) was inserted into the pAAV-U6-shRNA/spgRNA v2.0-CMV-EGFP-WPRE vector and packaged into the AAV2/9 virus and named as AAV-Gnao1-shRNA. By the way, the knockdown efficiency of the shRNA loaded with the virus has been verified by in vitro experiments, and its sequence is as following: 5′-GCGTGGAGTATGGTGACAA-3′. Viruses loaded with non-targeted gene sequences (scrambled sequence) were also constructed as negative controls (AAV-NC). To generate the Gnao1 overexpression AAV, the mouse Gnao1 coding sequence (1065 bp, NM_010308.3) was cloned into the pAAV-CMV-EGFP-P2A-3xFLAG-WPRE vector, which was then packaged into the AAV2/8 virus and called as AAV-Gnao1-OE. The virus construction and packaging were performed by OBiO Biology (Shanghai, China). The 6 μl virus of at titer of 10^12^ TU/ml was injected into the sciatic nerve via the epineurium or 10 μl virus at titer of 10^12^ TU/ml was intrathecally injected into the spinal cord with a micro-syringe as described before [20, 51].

### Establishment of an in vivo model of re-myelination

Eight-week-old adult mice were under deep anesthesia with 3% isofluorane and exposed the sciatic nerve of their left leg. The nerves were crushed with a fine hemostat for 30 s to establish a model of the myelin sheath regeneration. In addition, sham-operated mice were set up to expose their sciatic nerve without damaging nerve tissue. During the surgery and recovery from anesthesia, the body temperature of the mice were maintained with heating pads. After they are fully awake, the animals are kept in cages (3 in each cage) and are raised and fed as usual.

### Gait analysis

The gait parameters of mice were recorded using the CatWalk system (Noldus Information Technology, Netherlands), and analyzed with the software provided by the manufacturer. Mice were studied over a 3-week period (1, 4, 7, 10, 14, and 20 days) after surgery (n = 5 per group), the footprint, contact intensity, and sciatic nerve function index (SFI) were calculated and compared with control by the one-way ANOVA analysis followed by the Tukey’s multiple comparisons test.

### Cell culture

Primary SCs were isolated from neonatal rat (1–2 days) sciatic nerve as previously described. In brief, following the treatment with 1% collagenase (Gibco, Carlsbad, CA, USA) and 0.125% trypsin enzymes (Gibco), cell mixtures were resuspended in DMEM (Gibco) containing 10% fetal bovine serum (FBS; Gibco), penicillin–streptomycin (PS; Thermo Fisher Scientific, Cleveland, OH, USA) and 10 mM cytosine arabinoside (Sigma, St Louis, MO, USA), seeded in 50 μg/ml poly-D-lysine (PDL; Sigma) coated dishes and cultured for 1 day to remove fibroblasts. After that, the medium was changed to DMEM supplemented with 5 μm forskolin (Sigma), 2 ng/ml Neuregulin-1 (NRG1; R&D, MN, USA) and 10% FBS and continuous culture for 2 days. Cells were incubated with anti-Thy1 antibody (1:1000; Sigma) to further remove the remaining fibroblasts. The purity of SC was evaluated by immunostaining with S100β antibody (a SC marker; 1:500; Sigma). The second generation SC with purity > 95% was used in cell experiments.

DRG neurons were obtained by culturing and purifying isolated embryonic 15-day-old rat DRGs in 3 different mediums in PDL-coated dishes for 2–3 weeks. The order of application and acting time of the 3 mediums are as follows: First, DRGs were cultured using DMEM-HG medium (Gibco) containing 10% FBS for 1 day to ensure DRG adherence to the dish. Then, the neurobasal medium (NB; Gibco) supplemented with 50 ng/ml NGF (Sigma), 2% B27 (Gibco) and 2 mM L-glutamine (Gibco) was used for 2 days to promote the neurite growth. After that, the medium was changed to purification medium consisting of NB medium, uridine (Sigma) and 5-fluorodeoxyuridine (Sigma) to remove non-neuronal cells, and high purity DRG neurons were obtained after 3 times of circulating purification. DRG neurons were mainly used to assemble axon membranes for assaying the SC proliferation and migration.

### Small interfering RNA transfection

The Gnao1-shRNAs (RiboBio, Guangzhou, China) and their non-targeting negative control (NC or Scramble), at a 10 nM concentration, were transfected into SCs by using the transfection reagent RNAiMAX (Invitrogen, Carlsbad, CA, USA), according to the manufacturer’s protocol. Three siRNA sequences designed for Gnao1 were as following: #1, 5′-GCGTGGAGTATGGTGACAA-3′, #2, 5′-GGAAGGCAGACTCCAAGAT-3′, and #3, 5′-CCCACTTCACCTTCAAGAA-3′. Two days post-transfection, the knockdown efficiencies for siRNAs were evaluated with quantitative real-time PCR (qRT-PCR) and western blotting (WB), and the results were provided in supplementary Fig. 1 (Additional file 1: Fig. S1). The “#1” with the highest knockdown efficiency was selected for in vitro studies of SC functions such as migration, proliferation and differentiation, as well as for construction of AAV-Gnao1-shRNA.

### Cell proliferation assay

The proliferation of SCs cultured on two substrates, i.e. fascicular DRG neurites and PDL, was examined by using the 5-Ethynyl-2′-deoxyuridine (EdU) label kit (Ribobio). In brief, SCs (1 × 10^5^) were treated with 50 µM EdU for 24 h, and EdU and Hoechst 33342 staining was performed according to the manufacturer’s protocol after fixation with 4% PFA. After capturing 10 randomly selected areas per well using Fluorescence microscope (Leica), cell proliferation rates were calculated (EDU-positive cell number /Hoechst-labeled cell number, multiplied by 100%). Experiments were carried out in quadruplicate or in sevenfold.

### Cell migration assay

SC migration was evaluated with (1) cell scratch assay and (2) cell spheroid migration assay. Cell scratch assay was routinely measured by using the culture insert (Ibidi, DE, Germany). Briefly, SCs (5 × 10^3^) were seeded into culture inserts, which were placed into the 24-tissue-culture wells contained 500 μl DMEM supplement with 10% FBS. After incubation at 37 °C for 24 h, two separate cell islands of were obtained by removing the inserts, and then added mitomycin C (2 μg/ml; Sigma) into medium for another 12 h-incubation. Afterwards, the cell migration distance was measured using Image J in four independent experiments. In the cell spheroid migration assay, the physiological conditions were mimicked by co-culturing reaggregated SCs and fasciculated DRG axons. After SC reaggregates were obtained by inoculating SCs on a non-permissive substrate overnight and gentle shaking them every 2 h, they were seeded onto the fasciculated axons and cultured for 12 h to measure the distance that individual SCs migrate out of the reaggregates along the axons in seven independent experiments.

### SC differentiation assay

SCs were induced for 3 days by exposure to cAMP's membrane permeability analogs, the dibutyryl cyclic AMP (db-cAMP; Sigma), to acquire a differentiation phenotype, as previously described [51]. Briefly, SCs were cultured in proliferative medium (DMEM with 10% FBS and 20 ng/ml Nrg1) for 24 h, then the medium was replaced with non-proliferation medium (DMEM/F12 supplement with 1% FBS and 20 ng/ml Nrg1), 1 mM db-cAMP was added to initiate SC differentiation. The expression of markers of myelinating such as MAG (myelin-associated glycoprotein, MAG) were analyzed for up to 3 days.

### Immunohistochemistry (IHC) and immunocytochemistry (ICC)

Mice were perfused intracardially with 4% paraformaldehyde (PFA) and the PFA-post-fixed tissues were cryoprotected in optimal cutting temperature compound (OCT) and processed for 12 µm cryosections for IHC. ICC is used for PFA-fixed cells. Sections or PFA-fixed cells were permeated in 0.2% Triton-X100 in phosphate buffered saline (PBS), blocked with 5% donkey serum for 1–2 h at room temperature (RT), and incubated overnight at 4 °C with primary antibodies. Sections or cells were then washed thrice before corresponding fluorescence-conjugated secondary antibody (1:1000; Jackson Immunoresearch, PA, USA) incubation for 1 h at RT, following by three washes and mounting with coverslips. Images were captured using a fluorescence microscope (Leica). Primary antibodies used are: Mouse anti-S100β (1:200; Sigma, S2532), Rabbit anti-MAG (1:100; Invitrogen, 34–6200), Mouse anti-Gnao1 (1:1000; Santa Cruz, CA, USA, sc-13532), Rat anti-F4/80 (1:200; Abcam, ab6640), Mouse anti-β-tubulin-III (1:1000; Sigma, T8578).

### Western blotting (WB)

Cells or minced tissues were lysed in RIPA buffer (Beyotime, Shanghai, China) and centrifuged at 13,200 rpm for 15 min at 4 °C. After the quantification of protein concentration (Beyotime), WB was performed by following the same procedure described previously, except for the primary antibodies: Rabbit anti-MAG (1:100; Invitrogen, 34-6200), Mouse anti-Gnao1 (1:1000; Santa Cruz, sc-13532), Rabbit anti-Gnao1 (1:1000; Abcam, ab154001), Rabbit anti-AKT (1:1000; CST, 9271S), Rabbit anti-pAKT (1:1000; CST, 9272S), Rabbit anti-ERK1/2 (1:1000; CST, 9102S), Rabbit anti-pERK1/2 (1:1000; CST, 9101S), Rabbit anti-PI3K (1:1000; CST, 4292), Rabbit anti-pPI3K (1:1000; CST, 17366), and Mouse anti-GAPDH (1:50,000; Proteintech, Chicago, USA, 60004-1-Ig).

### Transmission electron microscopy (TEM)

The nerve tissues were embedded into Epon 812 epoxy resin after being fixed with 2.5% glutaraldehyde and post-fixed with 1% osmium tetroxide, and then ultrathinly cut into 50 nm thick sections. Thin sections were stained with lead citrate and uranyl acetate, and then viewed on a JEOL (JEM-1400) transmission electron microscope. Images were recorded with a digital camera and used to quantify axon diameter, myelin sheath thickness and layers, as well as *G* ratio, etc.

### In vitro cAMP measurement

The concentration of cAMP in the SCs transfected with NC or Gnao1-shRNA was measured using cAMP ELISA kit (NewEast Biosciences, Malvern, PA, #80203) according to the manufacturer’s instructions. Briefly, cells were homogenized in 0.1 M HCl plus 0.1% Triton X-100 for 10 min, and the clarified supernatant was obtained after centrifugation at 600 g for 5 min. For cAMP measurement, the 100 μl supernatant was diluted in a manufacturer-provided buffer, and added into the appropriate wells which coated mouse monoclonal antibody to cAMP, and the plate was incubated at room temperature on a plate shaker at 250 rpm for 2 h. After 3 washes with washing buffer, the substrate solution was added and incubated for 30 min without shaking. The optical density was read at 590 nm on a Microplate reader (Thermo Scientific), and the cAMP concentrations were quantified according to the standard product curve.

### RNA sequencing and data analysis

Total RNA from pre-and post-differentiated SCs transfected with NC or Gnao1-siRNA were extracted using TRIzol (Invitrogen). RNA-seq libraries were prepared using the VAHTS Universal V6 RNA-seq Library Prep Kit and sequenced on an Ilumina Novaseq 6000 platform by OE Biotech, Inc., Shanghai, China. RNA-seq reads were cleaned and mapped to the reference genome (mRatBN7.2) using HISAT2. FPKM and the read counts of each gene were obtained by HTSeq-count. Differential expression analysis was performed using the DESeq2, and Q value < 0.05 and fold change > 2 or fold change < 0.5 was set as the threshold for significantly differential expression genes (DEGs). Hierarchical cluster analysis of DEGs was performed using R (v 3.2.0) to demonstrate the expression pattern of genes in different groups and samples. Gene ontology (GO) and Kyoto Encyclopedia of Genes and Genomes analysis (KEGG) were performed using the OECloud tools at https://cloud.oebiotech.com/task/.

### Statistical analysis

Statistical analyses were done using GraphPad Prism 8.00 (http://www.graphpad.com). The two-tailed or one-tailed unpaired student's *t*-test was used to determine the significance between two samples. One-way analysis of variance (ANOVA) with Tukey's post-hoc for multiple comparisons was used to determine the significances for more than three samples. Quantitative was performed in a double-blind manner from at least three independent repeated experiments, and n value was the number of replicates. Data are shown as mean ± SEM in dot plots or bar graphs, and *P* < 0.05 was considered statistically significant and indicated in the figures with the following conventions: *P < 0.05, ** P < 0.01, and *** P < 0.001.

## Results

### Screening for novel myelination‑related G proteins and GPCRs from the transcriptomic data of SCs at various stages of myelination

**Fig. 1:**
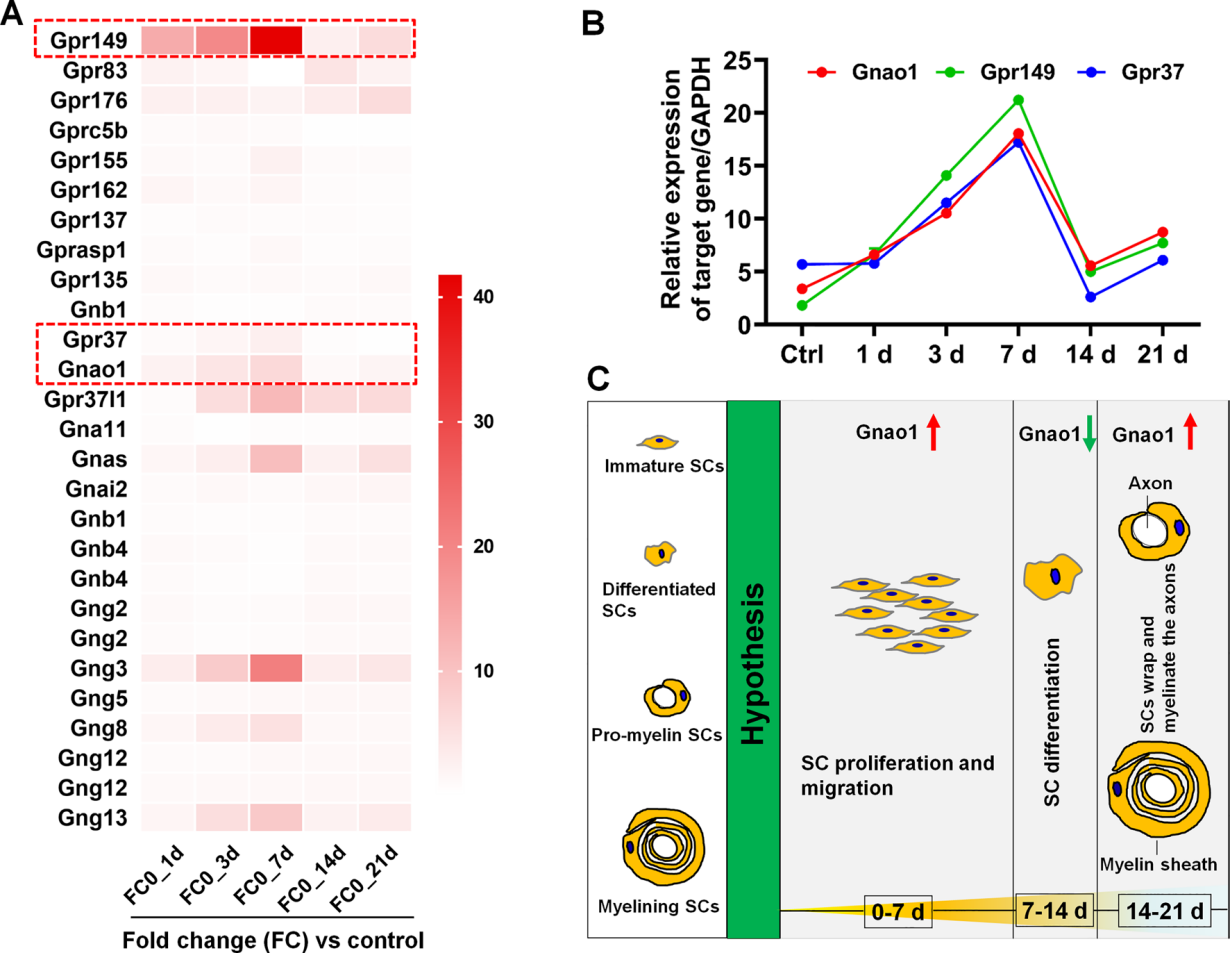
Screening of novel myelination-related G proteins and GPCRs and hypothesis of their regulation of myelination. **A** Heat map of the changes in the expression of G proteins and GPCRs during SC myelination (columns). The red dot box indicates 3 genes (GPR149, GPR37 and Gnao1) with the same expression trend. **B** QPCR analysis of the expression dynamics of 3 genes (GPR149, GPR37 and Gnao1) in SCs during myelination. n = 3. **C** The schematic diagram showing the possible mechanisms of Gnao1 in regulating SC myelination inferred from data analysis and co-expression assays, that is, on the initial stage of myelination (i.e., the preparation stage), the up-regulation of Gnao1 expression (red up arrow) may promote immature SC proliferation and migration; while the subsequent down-regulation of Gnao1 (green down arrow) may be related to SC differentiation, indicating entry into the pre-myelination stage. With the completion of SC differentiation, the expression of Gnao1 rose again, at which time the mature SCs began to wrap the axons and form myelin sheaths

To search for the novel G proteins and GPCRs involved in SC development and PNS myelination, we first analyzed the transcriptomic data (GSE163132) of SCs at various stages of myelination (i.e., immature, pre-myelination, and myelination), which was obtained by microarray analysis of SCs dissociated from the DRG neuron-SC co-culture system by using the laser capture microdissection at days 0, 1, 3, 7, 14, and 21 [51]. The 3,735 differentially expressed genes (DEGs) proteins were identified with a fold change (FC, mRNA levels at each time point versus the control) > 1.5 or < 0.67 and an adjusted *p*-values < 0.05 as screening conditions (Additional file 2: Table S1.1). Among the DEGs, we achieved 12 DEGs encoding G proteins, including 4 encode for Gα-subunits (i.e. Gnao1 for Gαo, Gnai2 for Gαi, Gnas for Gαs, and Gna11for Gα11); 2 genes (Gnb1 and Gnb4) for Gβ-subunits, as well as 6 genes (Gng2, Gng3, Gng5, Gng8, Gng12 and Gng13) for Gγ-subunits. Also, we obtained the 11 GPCRs including GPR37, GPR149, GPR83, and GPR155, among others (Additional file 2: Table S1.2). Based on the co-expression analysis, we found that Gnao1, Gnas, Gng 3, Gng 8, Gng 13, GPR37, and GPR149 showed similar dynamics, which is, compared with the control, their expression gradually increased up to 7 days, followed by a drastic decrease and reached the nadir at 14 days, and then rose again (Fig. 1A). In addition, we quantified the expression levels of Gnao1, GPR37, and GPR149 during SC myelination by RT-qPCR, showing that their expression increased in the early stage (days 1–7) and then up-regulated again after reaching the lowest level on days 14 (Fig. 1B), consistent with the above data analysis. GPR37 and GPR149 have been shown that negatively regulates CNS myelination by controlling the transition from early-differentiated to mature OLs [45, 50]. Therefore, we hypothesized that Gnao1 may be a negative regulator of myelination just like GPR37 and GPR149, and speculated that Gnao1 level dynamics in SCs during myelination might be responsible for its different functions, that is, on the initial stage of myelination (i.e., the preparation stage), the up-regulation of Gnao1 expression may promote immature SC proliferation and migration, and the subsequent down-regulation of Gnao1 may be responsible for SC differentiation, indicating SCs entry into the pre-myelination stage. With the completion of SC differentiation, the expression of Gnao1 rose again, at which time the mature SCs began to wrap the axons and form myelin sheaths (Fig. 1C). In the following section, we were sought to investigate and explain the roles of Gnao1 in the PNS myelination.

### Endogenous Gnao1 alterations in SCs affect re-myelination, but Gnao1 in neurons not

To assess the in *vivo* contribution of Gnao1 to PNS myelination, we first analyzed its mRNA and protein levels in nerve tissue (sciatic nerve and spinal cord) and myelination-related cells (neurons and SCs) by RT-PCR and WB analysis, and found that Gnao1 is expressed in nerve tissue, SCs and neurons (Additional file 1: Fig. S1), which is consistent with previous reports [5]. In view of this, we next sought to alter Gnao1 levels in spinal cord (neurons) or sciatic nerve (SCs) by intrathecal injection or sciatic nerve in situ injection of viruses carrying the Gnao1 interfering (Gnao1-shRNA, its knockdown efficiency has been determined by qPCR and WB as shown in Additional file 1: Fig. S2) and coding sequences (Gnao1-OE), respectively, to generate mice with Gnao1 knockdown or overexpression in the sciatic nerve (hereafter referred to as Gnao1-NKD and Gnao1-NOE), as well as mice with Gnao1 knockdown in spinal cords (hereafter named Gnao1-SKD). WB and IHC analysis of the sciatic nerves of Gnao1-NKD and -NOE mice (Additional file 1: Fig. S3), as well as the spinal cords of Gnao1-SKD mice (Additional file 1: Fig. S4), revealed that in these mice, the injected virus effectively up-regulated or down-regulated the protein levels of Gnao1. Notably, in Gnao1-NKD and Gnao1-NOE mice, knockdown or overexpression occurred mainly in the SCs of the sciatic nerves, whereas in Gnao1-SKD mice, such changes were mainly seen in spinal cord neurons, even the viruses do not have no SC- or neuron-specific promoters (Additional file 1: Figs. S3−34).

**Fig. 2:**
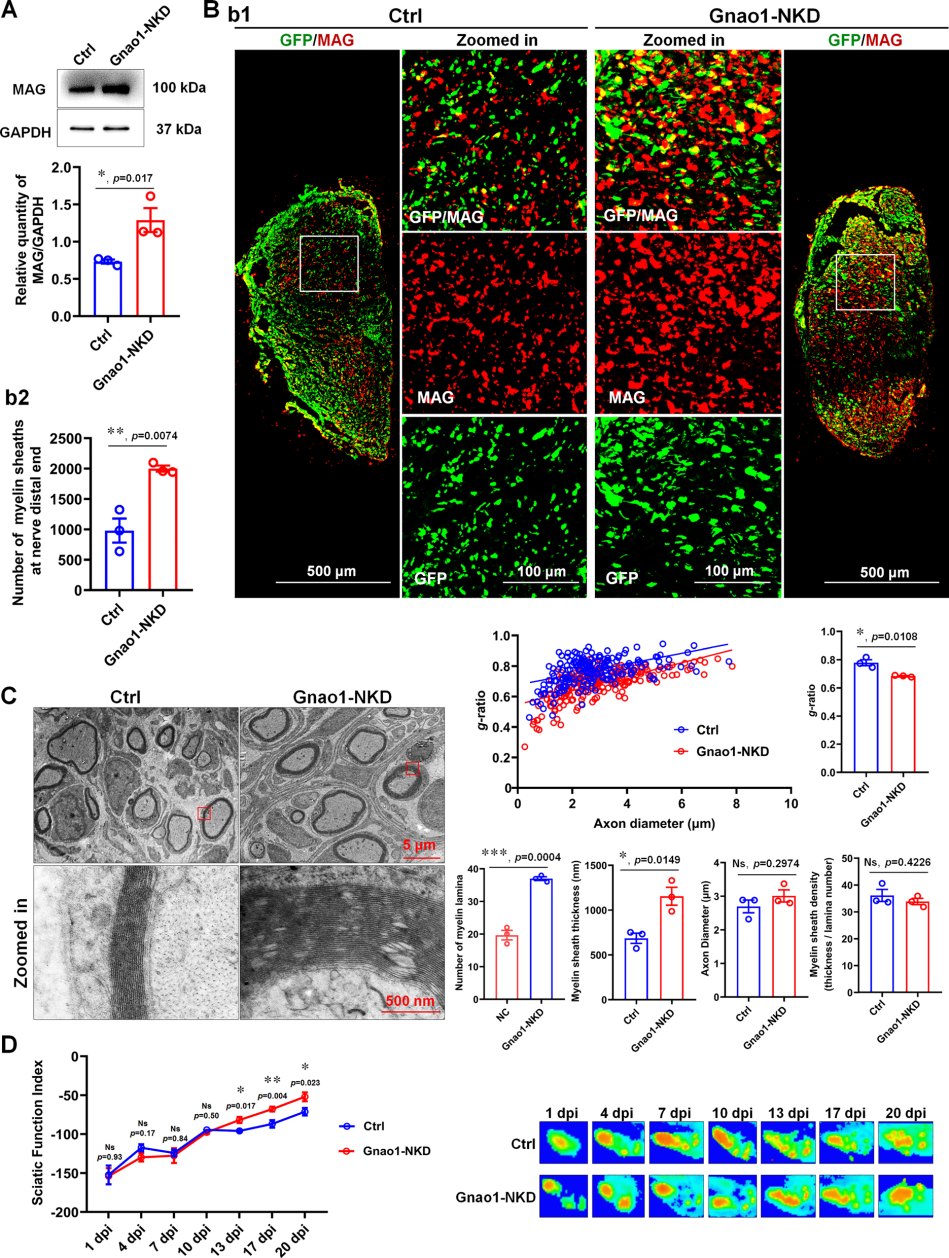
Knockdown Gnao1 expression in in SCs of sciatic nerve promotes myelin re-formation after nerve injury. **A** The expression of MAG in the regenerated nerves of Gnao1-NKD mice and controls (Ctrl) was detected by WB. As shown are representative WB images (up) and the statistical histogram (bottom). *T*-test, n = 3, *, *p* < 0.05 vs control. **Bb1** IHC images of regenerative nerves from Gnao1-NKD mice and controls, MAG for regenerative myelin-associated protein (red), GFP for viral expression (green), scale bar = 500 μm. Zoomed in is the enlargement of the white box area, scale bar = 100 μm. **Bb2** The histogram is a quantitative statistic of the amount of myelin sheaths per unit area. *T*-test, n = 3, **,* p* < 0.01 vs control. **C** Representative transmission electron microscopy (TEM) images of the regenerated sciatic nerve of Gnao1-NKD mice and controls after 21 day injury, showing the myelin sheath structure (left), scale bar = 5 μm. Zoomed in is the magnification of the red box, scale bar = 500 nm. The distribution map of the *g*-ratio of myelin sheath of the regenerated nerve in Gnao1-NKD mice and controls and the diameter of the regenerated axon wrapped by myelin sheath (middle top), and the histogram are the quantitative statistics of *g*-ratio (upper right) myelin thickness, lamellar number, axon diameter and myelin density (lower right). *T*-test, n = 3, Ns, *p* > 0.05 vs control, no statistical difference. *, *p* < 0.05, ***, *p* < 0.001 vs control. **D** CatWalk gait analysis performed at 1, 4, 7, 10, 13, 17 and 21 days post-injury, the left histograms showing the values of sciatic function index (SFI) and representative footprints of the left hind paw (injured side) of Gnao1-NKD mice and controls are showed on the right. *T*-test, n = 3, Ns, *p* > 0.05 vs control, no statistical difference. *, *p* < 0.05, **, *p* < 0.01 vs control

Having established that virus administration effectively alters Gnao1 in spinal cords and nerves, we next use a sciatic nerve crush model (i.e., an in vivo re-myelination model) to determine whether Gnao1 is required for re-myelination after injury. To this end, we crushed the sciatic nerves of Gnao1-NKD, -NOE and -SKD mice, together with their respective control, and analyzed the nerves at 28 days post injury (dpi), a time point when the myelin debris have been cleared and re-differentiated SCs are intensely re-myelination the regenerated axons [22]. WB and IHC staining showed that, MAG, a myelin structural protein, was expressed at significantly higher levels in the sciatic nerve of Gnao1-NKD mice than in their controls, and GFP staining again revealed a strong infection efficiency of the virus in injected animals. More importantly, we never observed GFP in SCs that have re-myelinated (Fig. 2A, B). Ultrastructural analyses of myelin sheaths by TEM showed that there were more myelinated nerve fibers observed in the Gnao1-NKD mice, and the myelin layers and thickness were larger than those of control mice.* G*-ratio analysis further proved that the re-myelinated axons in Gnao1-NKD mice have thicker myelin (i.e., lower *g*-ratios) compared with controls (Fig. 2C), which indicated that the onset of re-myelination in the sciatic nerves from Gnao1-NKD mice was accelerated. Given that re-myelination is associated with animal motor function, we evaluated the gait dynamics of Gnao1-NKD and its control mice. The Gnao1-NKD mice showed more normality in walking than control as evidenced by the sciatic functional index (SFI) scores and paw area (Fig. 2D). These data suggest that reduction of Gnao1 in SCs is beneficial to re-myelination of injured axons.

**Fig. 3:**
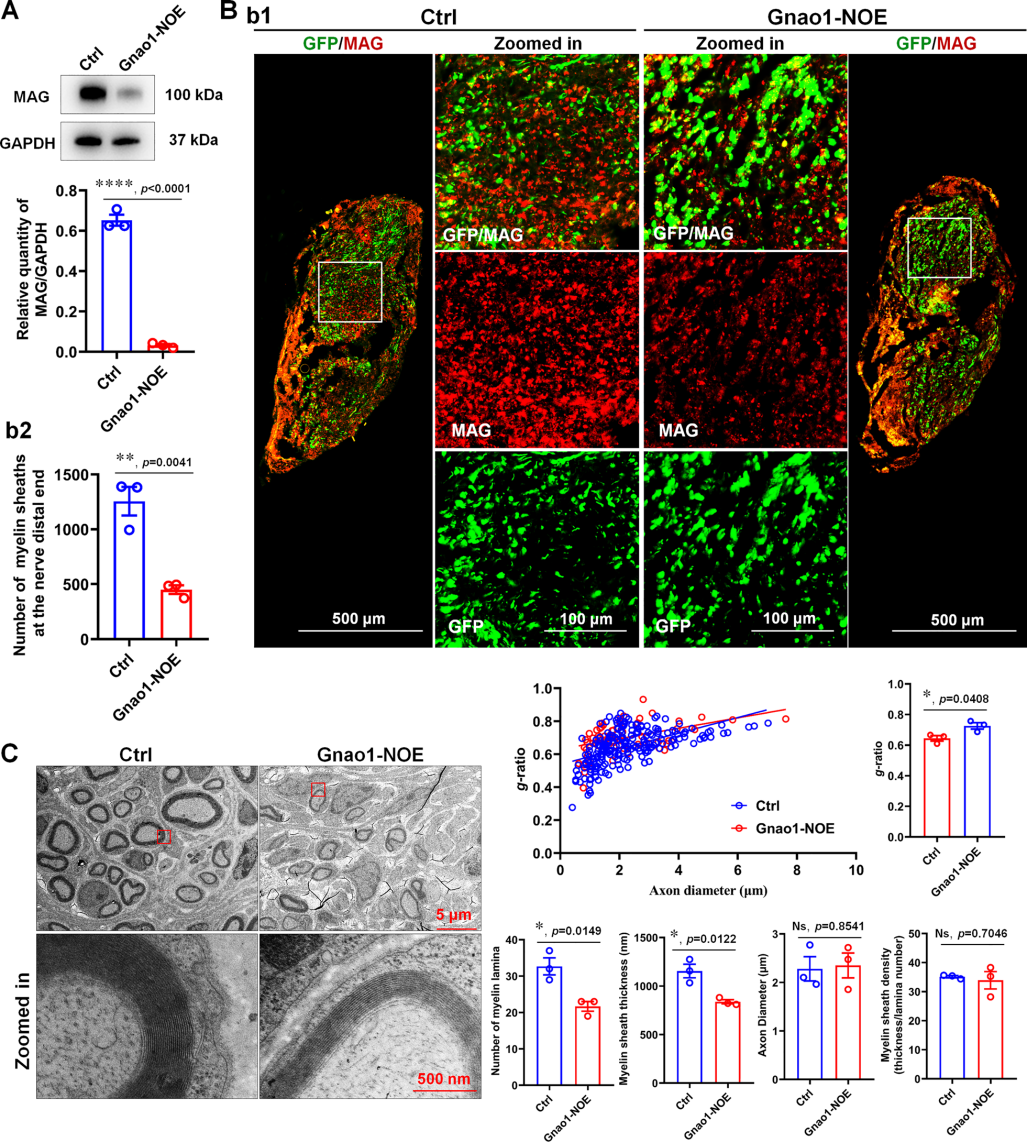
Overexpression Gnao1 level in SCs of sciatic nerve inhibits myelin re-formation after nerve injury. **A** The expression of MAG in the regenerated nerves of Gnao1-NOE mice and controls (Ctrl) was detected by WB. As shown are representative WB images (up) and the statistical histogram (bottom). *T*-test, n = 3, ****, *p* < 0.0001 vs control. **Bb1** IHC images of regenerative nerves from Gnao1-NOE mice and controls, MAG for regenerative myelin-associated protein (red), GFP for viral expression (green), scale bar = 500 μm. Zoomed in is the enlargement of the white box area, scale bar = 100 μm. **Bb2** The histogram is a quantitative statistic of the amount of myelin sheaths per unit area. *T*-test, n = 3, **,* p* < 0.01 vs control. **C** Representative transmission electron microscopy (TEM) images of the regenerated sciatic nerve of Gnao1-NOE mice and controls after 21 day injury, showing the myelin sheath structure (left), scale bar = 5 μm. Zoomed in is the magnification of the red box, scale bar = 500 nm. The distribution map of the *g*-ratio of myelin sheath of the regenerated nerve in Gnao1-NOE mice and controls and the diameter of the regenerated axon wrapped by myelin sheath (middle top), and the histogram are the quantitative statistics of *g*-ratio (upper right) myelin thickness, lamellar number, axon diameter and myelin density (lower right). *T*-test, n = 3, Ns, *p* > 0.05 vs control, no statistical difference. *, *p* < 0.05 vs control

To more conclusively demonstrate that the downregulation of endogenous Gnao1 in SCs contributes to re-myelination, we next observed the ability of SCs by analyzing the regenerated sciatic nerves from Gnao1-NOE mice (i.e., mice with overexpressing Gnao1 in SCs) and controls. As expected, the re-myelination is significantly impaired in sciatic nerves from Gnao1-NOE mice based on MAG levels assessed by WB and IHC (Fig. 3A, B). TEM analyses further revealed many deficits at the ultrastructural level in regenerated nerves of Gnao1-NOE mice at 28 dpi, including significantly more unmyelinated large-caliber axons (> 1 μm), abnormal SC cytoplasmic protrusions and thinner myelin sheaths (i.e., higher *g*-ratios) compared with their control animals (Fig. 3C). Our data so far further suggest that Gnao1 alterations in SCs do affect re-myelination.

**Fig. 4:**
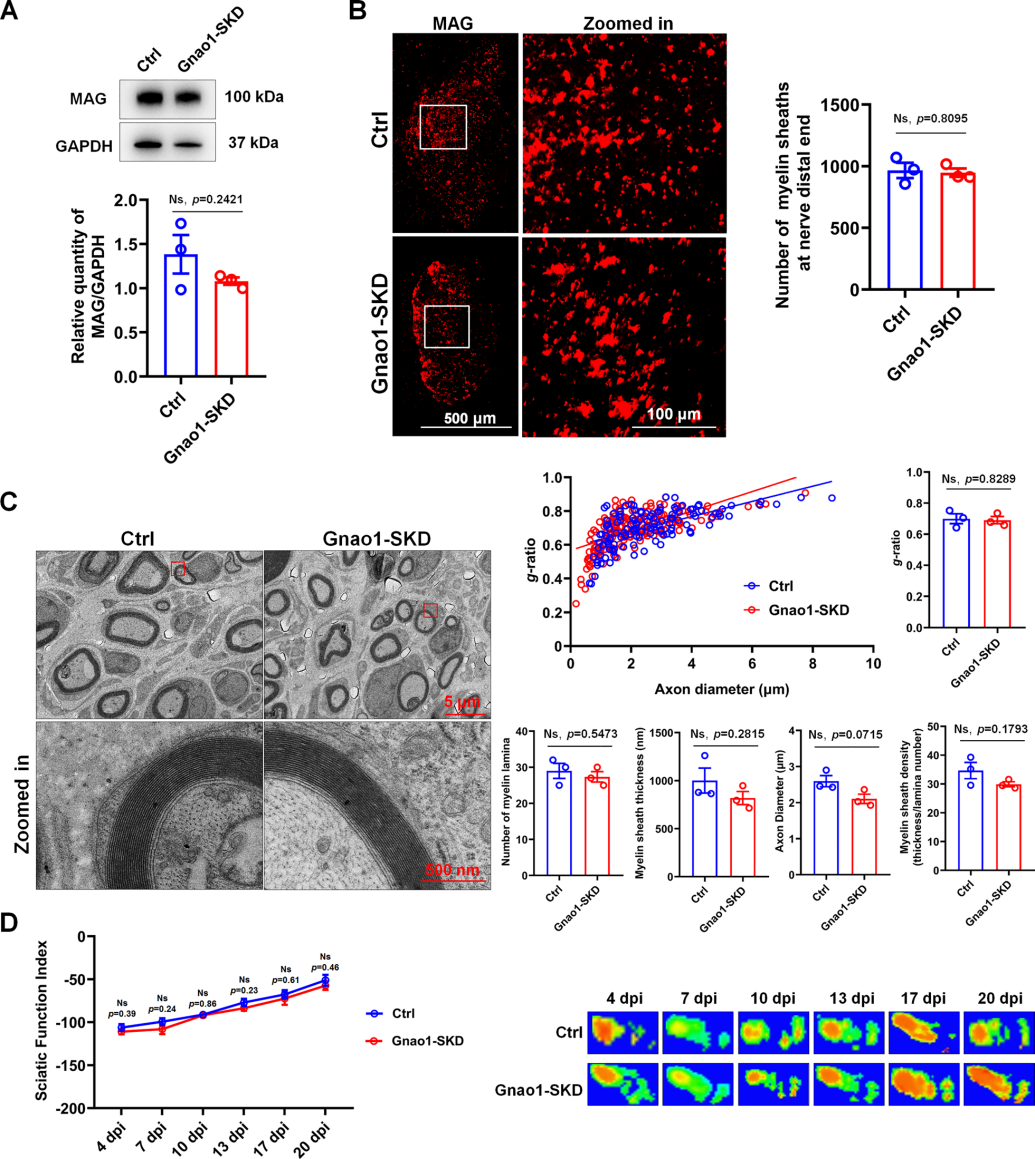
Knockdown Gnao1 expression in spinal cord neurons did not affect re-myelination after sciatic nerve injury. **A** The expression of MAG in the regenerated nerves of Gnao1-SKD mice and controls (Ctrl) was detected by WB. As shown are representative WB images (up) and the statistical histogram (bottom). *T*-test, n = 3, Ns, *p* > 0.05 vs control, no statistical difference. **B** IHC images of regenerative nerves from Gnao1-SKD mice and controls, MAG for regenerative myelin-associated protein (red), scale bar = 500 μm. Zoomed in is the enlargement of the white box area, scale bar = 100 μm. The histogram is a quantitative statistic of the amount of myelin sheaths per unit area. *T*-test, n = 3, Ns, *p* > 0.05 vs control, no statistical difference. **C** Representative transmission electron microscopy (TEM) images of the regenerated sciatic nerve of Gnao1-SKD mice and controls after 21 day injury, showing the myelin sheath structure (left), scale bar = 5 μm. Zoomed in is the magnification of the red box, scale bar = 500 nm. The distribution map of the *g*-ratio of myelin sheath of the regenerated nerve in Gnao1-NKD mice and controls and the diameter of the regenerated axon wrapped by myelin sheath (middle top), and the histogram are the quantitative statistics of *g*-ratio (upper right) myelin thickness, lamellar number, axon diameter and myelin density (lower right). *T*-test, n = 3, Ns, *p* > 0.05 vs control, no statistical difference. **D** CatWalk gait analysis performed at 4, 7, 10, 13, 17 and 20 days post-injury, the left histograms showing the values of SFI and representative footprints of the left hind paw (injured side) of Gnao1-SKD mice and controls are showed on the right. *T*-test, n = 3, Ns, *p* > 0.05 vs control, no statistical difference

Given that myelination involves the interactions between SCs and neurons and that Gnao1 is expressed in neurons, we next observed whether Gnao1 changes in neurons affect re-myelination after nerve injury. We examined regenerated sciatic nerves of Gnao1-SKD mice (i.e., mice with Gnao1 knockdown in spinal cord neurons) and controls. We did not observe differences between the two groups in the levels of MAG by WB and IHC (Fig. 4A, B). Consistent with this observation, the ultrastructural analyses by TEM further demonstrated that there are no differences in myelin sheath ultrastructure or thickness in Gnao1-SKD mice versus controls (Fig. 4C). Finally, we compared movement properties in Gnao1-SKD and control mice, and found no difference in walking dynamics and paw area between the two groups (Fig. 4D). Altogether, these results suggest that knockdown of Gnao1 in spinal cord neurons does not affect re-myelination in sciatic nerves after injury.

Collectively, we could conclude that Gnao1 in SCs contributes to the re-myelination of nascent axons after peripheral never injury (PNI), but that Gnao1 changes in spinal cord neurons may not be involved in this process.

### Gnao1 knockdown inhibits SC proliferation and migration, but these effects are compensated or counterbalanced by signals from neurons

As it has been determined that only SC endogenous Gnao1 changes affect the re-myelination after PNI, we next sought to investigate the effects of Gnao1 on various biological behaviors of SCs during myelination, including proliferation, migration and differentiation. To this end, we used an Gnao1 knockdown approach in SCs, that is, SC culture were treated with Gnao1-shRNA or scrambled sequence (i.e., negative control, NC) for 48 h to obtain Gnao1-knockdown SCs (referred to as Gnao1-KD-SCs) and control (Additional file 1: Fig. S2) for the following in vitro experimental studies.

**Fig. 5:**
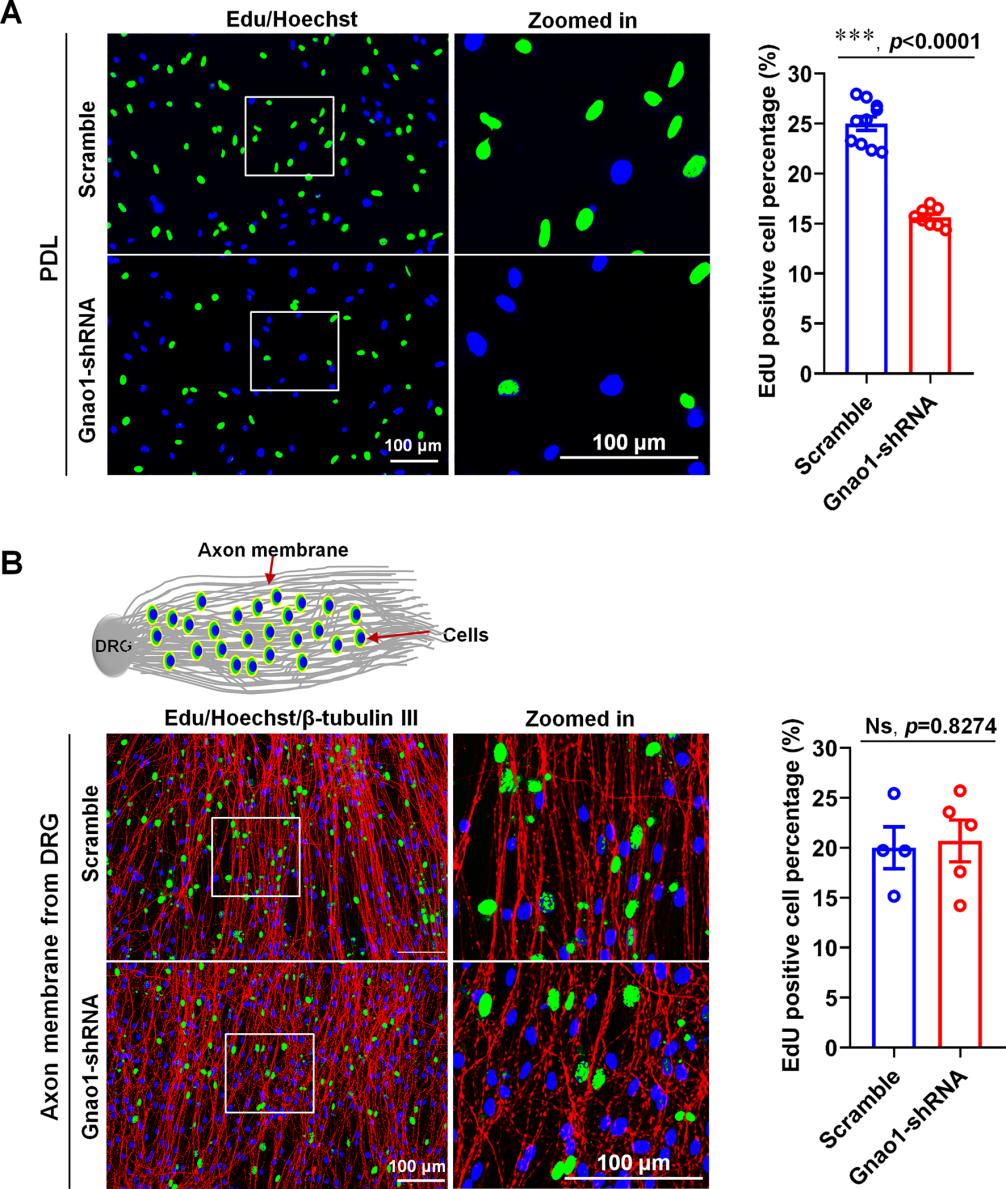
Gnao1 knockdown in SCs inhibited proliferation of SCs seeded on PDL but did not affect the proliferation of SCs seeded on fasciculated DRG axons. **A** The proliferation rate of Gnao1-shRNA or scramble-shRNA transfected SCs cultured on PDL are shown in the representative ICC images (left) and statistical analyses (right). In the ICC images, green dots (EdU positive), the proliferating SCs; blue dots (Hoechst), the nucleus, scale bar = 100 μm. Zoomed in is the magnification of the white box, scale bar = 100 μm. Histogram shows that Gnao1 knockdown significantly decreases SC proliferation. *T*-test, n = 9, ***, *p* < 0.0001 vs Scramble. **B** The proliferation rate of Gnao1-shRNA or scramble-shRNA transfected SCs cultured on fasciculated DRG axons are shown in the representative ICC images (left) and statistical analyses (right). In the ICC images, green dots (EdU positive), the proliferating SCs; blue dots (Hoechst), the nucleus, scale bar = 100 μm. Zoomed in is the magnification of the white box, scale bar = 100 μm. Histogram shows that Gnao1 knockdown did not affect SC proliferation. *T*-test, n = 4–5, Ns, *p* > 0.05 vs Scramble, no statistical difference

We studied the effect of Gnao1 deletion on SC proliferation using 5-Ethynyl-2’-deoxyuridine (EdU) incorporation assays, and noticed that the number of EdU-positive cells was significantly lower in Gnao1-KD-SCs compared to the negative control cells, suggesting that Gnao1 knockdown reduced SC proliferation (Fig. 5A). In addition, considering that under physiological conditions, signals from neurons affect SC proliferation, we thus observed the effect of Gnao1 on SC proliferation in a co-culture system of SCs (Gnao1-KD-SCs and control) with DRG neurons. Our results showed that, unlike previous findings, there are no differences in the number of EdU-positive cells between Gnao1-KD-SCs and control when cultured on the fascicular DRG neurites (Fig. 5B), presumably because some cytokines and neurotrophins released from neurons, such as the neuregulin-1 (NRG), have the powerful ability to promote cell proliferation, which compensated for the reduced cell proliferation caused by Gnao1 knockdown.

**Fig. 6:**
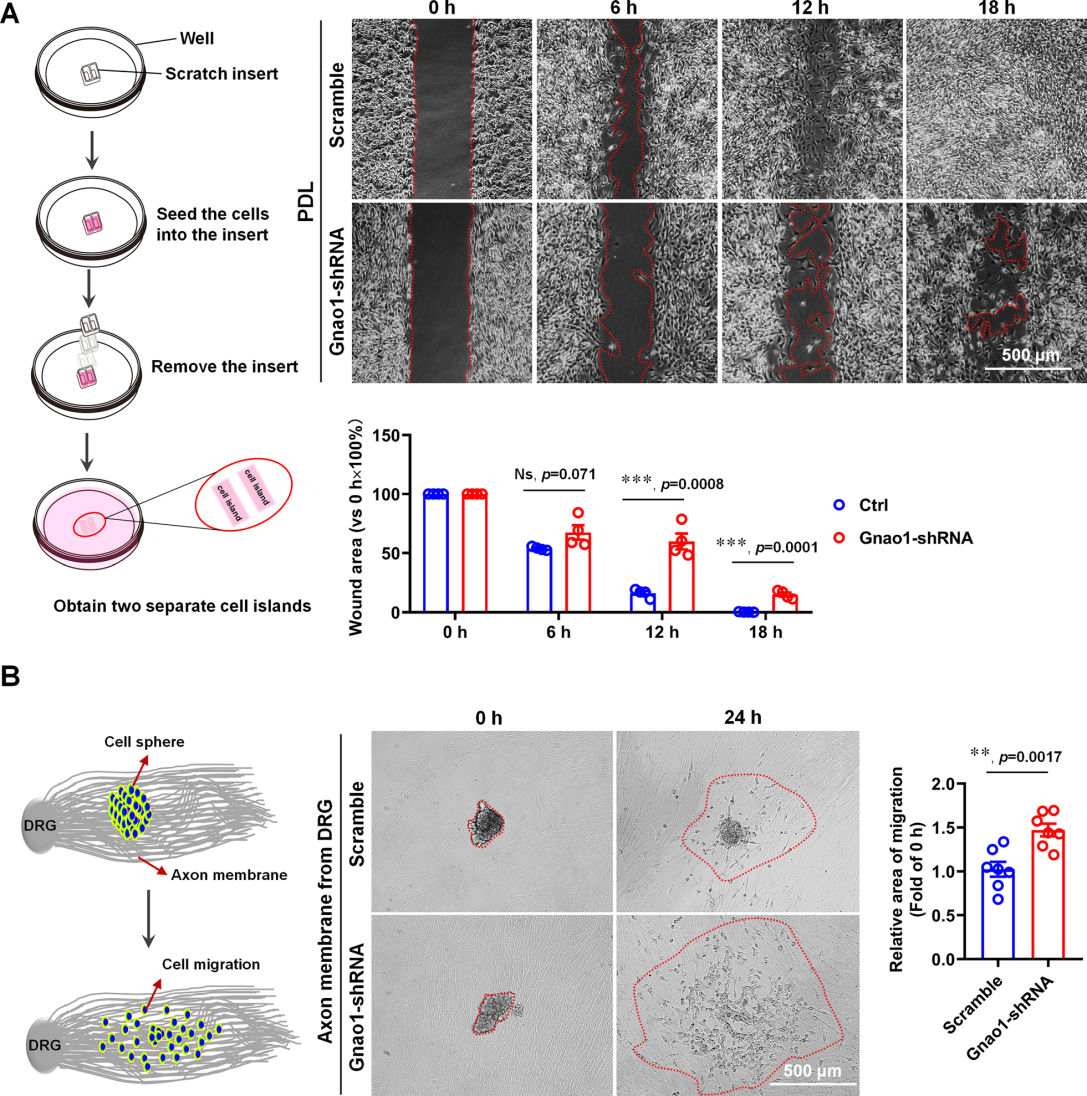
Down-regulation of Gnao1 expression in SCs reduced migration of SCs seeded on PDL (scratch migration assay), but resulted in increased migration when SCs are seeded on fasciculated DRG axons. **A** Results of an insert-based scratch migration assay. The left diagram shows the experimental process, and the right shows SCs transfected with Gnao1-shRNA reduced migration compared with that of SCs transfected with Scramble-shRNA. scale bar = 500 μm. The red dashed circle indicates the area of the scratch that has not healed. *T*-test, n = 4, Ns, *p* > 0.05 vs scramble, no statistical difference. ***, *p* < 0.001 vs scramble. **B** Results of a cell aggregation migration assay on the fasciculated DRG axons. The left diagram shows the experimental process, and the right shows that Gnao1 knockdown in SCs significantly increases SC migration from the SC aggregates on fasciculated DRG axons. Scale bar = 500 μm. Red dashed circles indicate the area of SC migration. *T*-test, n = 7, **, *p* < 0.01 vs Scramble

We performed the scratch assays and cell spheroid migration assays in the co-culture system (i.e., SC sphere co-cultured with fascicular DRG neurites) to determine the impact of Gnao1 knockdown on SC migration. As shown in scratch experiments (Fig. 6A), loss of Gnao1 results in a significantly slowing of wound healing, suggesting that deletion of Gnao1 inhibits migration of SCs cultured on PDL. Surprisingly, we obtained the exact opposite results in cell sphere migration experiments, that is, Gnao1 knockdown apparently promoted SC migration when the cell spheres were cultured on fascicular DRG axons (Fig. 6B), maybe due to various molecules from DRG neurons counteracting or even reversing the inhibitory effects on cell migration caused by Gnao1 knockdown.

Taken together, these data indicate that Gnao1 may act as a positive regulator of SC proliferation and migration, but that these effects are only seen in SC-alone culture (that is, cultured on PDL), while under the co-culture conditions (i.e., cultured on fascicular DRG neurites), those effects are compensated or counterbalanced by signals from neurons, such as NRG.

### Gnao1 deficiency promotes SC differentiation in vitro

**Fig. 7:**
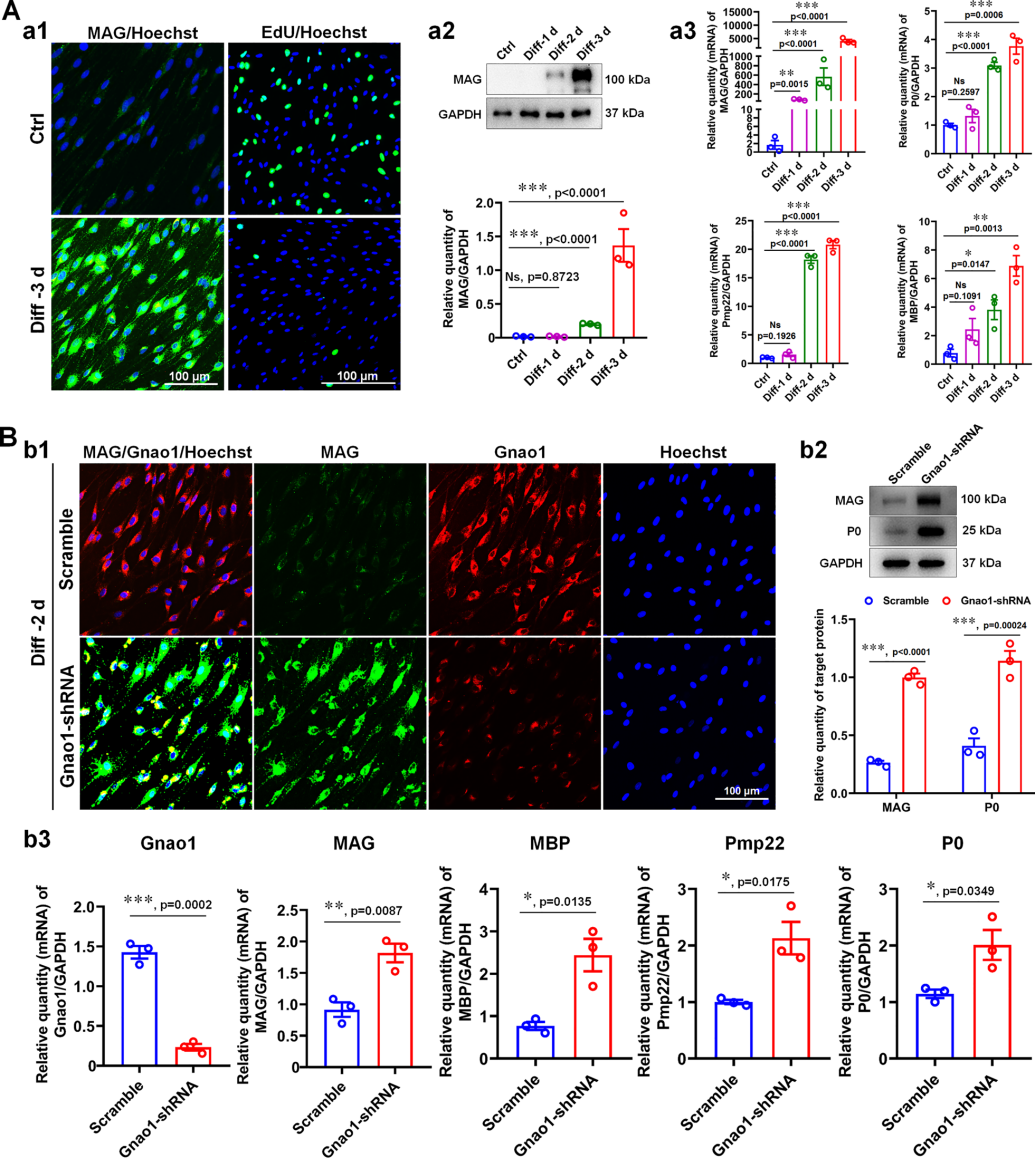
Down-regulation of Gnao1 expression in SCs accelerated SC differentiation. **A** Establishment of SC in vitro differentiation model. Immunocytochemical analyses of MAG and EdU label assay showed that MAG expression (green) increased and proliferation ability (green) decreased of 3 day-induction SCs with db-cAMP (**a1**), which was further proved by WB analysis (**a2**) and qPCR (**a3**), indicating that the in vitro differentiation model was successfully established. Scale bar in (**a1**) = 100 μm. *T*-test, n = 3, Ns, *p* > 0.05 vs controls, no statistical difference. *, *p* < 0.05, **, *p* < 0.01, and ***, *p* < 0.001 vs controls. **B** Gnao1 deletion in SCs accelerated SC differentiation. **Bb1** Representative immunocytochemical analyses of MAG (green) and Gnao1 (red) showed that the differentiation of SCs treated with Gnao1-shRNA or Scramble, indicating that knockdown Gnao1of SCs promote SC differentiation. Scale bar = 100 μm. **Bb2** WB comparing MAG and P0 levels in differentiated cells treated with Gnao1-shRNA or Scramble. Also shown are representative WB images, with GAPDH as the internal standard. *T*-test, n = 3, ***, *p* < 0.001 vs scramble. **Bb3** QPCR comparing mRNA levels of MAG, P0, MBP, PMP22 and Gnao1 in differentiated SCs treated with Gnao1-shRNA or Scramble. *T*-test, n = 3, *, *p* < 0.05, **, *p* < 0.01 and ***, *p* < 0.001 vs Scramble

To assess the influence of Gnao1 on SC differentiation, we made use of a classical SC in vitro differentiation model [28], in which, the 1 mM db-cAMP and 20 ng/ml HRG were used to induce SC differentiation. After 3 days of induction, the expression of MAG, a typical marker associated with myelinating SCs, was significantly elevated, accompanied by a dramatically decrease in the proliferative capacity of SCs, as shown by IHC and WB analysis (Fig. 7Aa1, a2). In addition, we also detected the dramatic increase of other markers that define the middle (MAG) and late (P0, PMP22, and MBP) phases of SC differentiation into myelin-forming cells by qRT-PCR (Fig. 7Aa3), indicating that SCs had reached a mature phenotype (i.e., differentiated SCs). Next, Gnao1-KD-SCs and control were stimulated with the above-described induction system, and the effects of Gnao1 on SC differentiation was detected. We found that the absence of Gnao1 increased the percentage of MAG-expressing cells, as evaluated by immune-labelling for MAG at 3 days of induction (Fig. 7Bb1). Furthermore, WB results showed that Gnao1-KD-SCs exhibited higher expression levels of MAG and P0 compared to those of the controls (Fig. 7Bb2). Similarly, the qPCR detection revealed that knockdown of Gnao1 in SCs also resulted in the increased expressions of P0, PMP22, and MBP by (Fig. 7Bb3). In summary, these results suggest that Gnao1 negatively regulates the differentiation of SCs in vitro.

Based on the data so far, we speculated that Gnao1 achieves regulation of PNS myelination probably by coordinating of SC proliferation, migration and differentiation, among which negative regulation of SC differentiation may be the key.

### Gnao1 deficiency in SCs result in the elevation of cAMP level and activation of PI3K-AKT cascade, contributing to SC differentiation

To investigate the underlying mechanism by which Gnao1 regulates SC differentiation, we performed RNA sequencing on Gnao1-KD-SCs and controls (NCs), as well as 2-day-differentiated Gnao1-KD-SCs and controls (Called as Diff-Gnao1-KD-SCs and Diff-NCs, respectively). By pairwise comparison of sequencing data together with their respective controls (cutoff: fold change (FC) > 1.5 or < 0.67 plus *p*-value < 0.05), we found that 841 differentially expressed genes (DEGs) were up-regulated and 1119 down-regulated DEGs in Gnao1-KD-SCs versus NCs, 1931 up-regulated and 1676 down-regulated DEGs in Diff-NCs compared with NCs, 2489 up-regulated and 2183 down-regulated DEGs in Diff-Gnao1-KD-SCs versus Gnao1-KD-SCs, as well as 34 up-regulated and 127 down-regulated DEGs in Diff-Gnao1-KD-SCs versus Diff-NCs (Additional file 1: Fig. S5A and Additional file 3: Table S2). KEGG analysis revealed that the common functions of the up-regulated DEGs in the 4 DEG datasets were related to cAMP signaling pathway and PI3K-AKT signaling pathway (Additional file 1: Fig. S5B-E and Additional file 3: Table S2), indicating that the two may be necessary for Gnao1 to regulate SC differentiation.

**Fig. 8:**
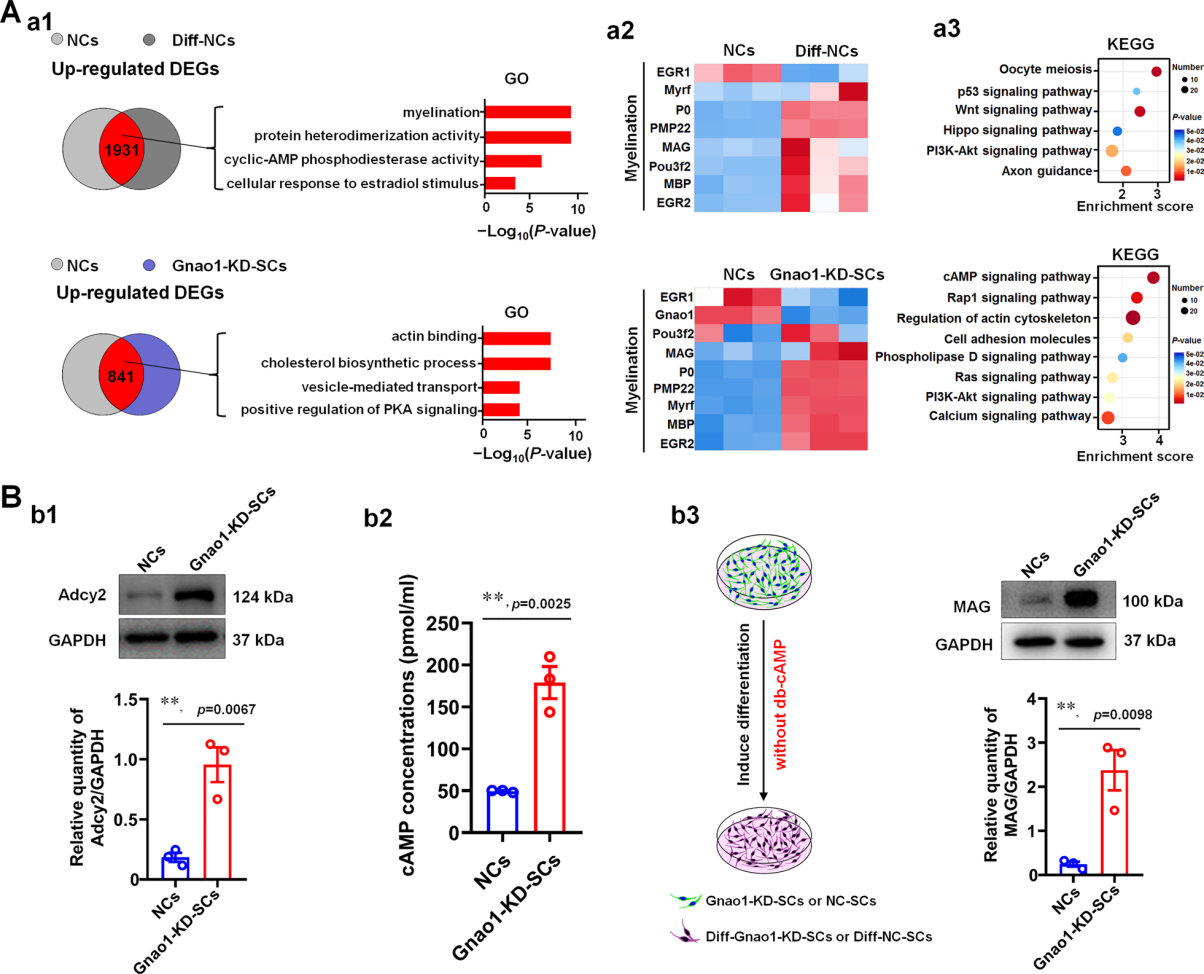
SCs lacking Gnao1 display more differentiation due to the elevated cAMP levels. **Aa1** Gene ontology analysis of the up-regulated differentially expressed genes (DEGs) in the differentiated SCs (Diff-NCs) and Gnao1-KD-SCs compared to controls (NCs). Bar graph showing representative GO categories of those DEGs. **Aa2** Heat map showing DEGs function related to myelination. **Aa3** KEGG analysis of the up-regulated DEGs in Diff-NCs and Gnao1-KD-SCs compared to NCs. Bubble diagram showing representative KEGG categories of those DEGs. **Bb1** WB analysis of Adcy2 in Gnao1-KD-SCs and controls (NCs), as shown are representative WB images (up) and the statistical histogram (bottom). *T*-test, n = 3, **, *p* < 0.01 vs NCs. **Bb2** The levels of cAMP in Gnao1-KD-SCs and NCs were detected by enzyme-linked immunosorbent assay (ELISA). *T*-test, n = 3, **, *p* < 0.01 vs NCs. **Bb3** Differentiation of Gnao1-KD-SCs and controls treated with db-cAMP free SC in vitro differentiation medium. WB analysis showing Gnao1-KD-SCs expressed higher level of MAG compared with NCs, indicating that Gnao1-KD-SCs could differentiate even in the absence of db-cAMP. Schematic display experimental method (left), also shown are representative WB images and quantitative statistics (right). *T*-test, n = 3, **, *p* < 0.01 vs NCs

More importantly, the above data analysis gave us an interest hint that knocking down Gnao1 expression in SCs would result in the change in levels of cAMP (an important second messenger that activate signaling affecting SC differentiation [15, 30]), which may be an important reason why we previously found that Gnao1-KD-SCs are more readily differentiated than normal SCs. To further demonstrate this, we focused on 2 sets of data, that is, 841 up-regulated DEGs between Gnao1-KD-SCs and NCs, as well as the 1931 up-regulated DEGs between Diff-NCs and NCs. Gene ontology and KEGG analysis revealed that functions of DEGs between Diff-NCs and NCs were associated with myelination, cAMP phosphodiesterase activity and cellular response to estradiol stimulus, consistent with the responses induced by cAMP mimics to stimulate SC differentiation. The functions of the DEGs between Gnao1-KD-SCs and NCs were related to cholesterol biosynthesis, actin binding, and activation of PKA signaling (Fig. 8Aa1, a3 and Additional file 3: Table S2), these are the events that occurred during SC myelination [39], indicating that knocking down Gnao1 expression in SCs favored for the differentiation of SCs. Moreover, we also noticed that the increased levels of DEGs in the 2 sets associated with myelination (Fig. 8Aa2), including myelin component proteins (MPZ, MBP, PMP22 and MAG), and positive myelin-related regulatory factors (Egr2, Myrf, Pou3f1 and Pou3f2). Remarkably, the inclusion of 2 additional sets of DEGs (i.e. 2489 up-regulated DEGs in Diff-Gnao1-KD-SCs versus Gnao1-KD-SCs and 34 up-regulated DEGs in Diff-Gnao1-KD-SCs versus Diff-NCs) corroborated these findings (Additional file 1: Fig. S6A).

We next proceeded with experiments to confirm the findings of the sequencing data analysis. First, we analyzed the expression of myelin-related factors, including myelin component proteins (MPZ, MBP, Pmp22 and MAG), regulatory factors (Egr2, Pou3f1, Pou3f2 and Id2), and the molecules that have been proven to affect myelination (Dok4 [2], Vamp7 [8], ErbB2/3) by qPCR, and identified their higher levels in Gnao1-KD-SCs, Diff-Gnao1-KD-SCs and Diff-NCs compared to NCs (Additional file 1: Fig. S6B). Notably, we found that the adenylate cyclase 2 (Adcy2), an essential cAMP signaling pathway component responsible for cAMP formation, was more highly expressed in the Gnao1-KD-SCs compared with control (Additional file 1: Fig. S6Bb3), suggesting that knockdown of Gnao1 in SCs leads to an increase in cAMP content. Motivated by the results, we next respectively evaluated the level of Adcy2 by WB and the content of cAMP by ELISA in Gnao1-KD-SCs and NCs, and found that down-regulation of Gnao1 expression in SCs resulted in the up-regulation of the level of Adcy2 (Fig. 8Bb1) and the elevating cAMP content (Fig. 8Bb2), which was in line with the previous report that Gnao1-encoded Gα0 protein can affect cAMP production in the cellular system [10]. As we known, in the in vitro SC differentiation model, the db-cAMP (cAMP mimics) were used to induce the differentiation of SCs. In addition, given that knockdown Gnao1 in SCs enhanced the cAMP content, we therefore wonder whether Gnao1-KD-SCs have capacity of differentiation without the addition of db-cAMP. To investigate this, we cultured Gnao1-KD-SCs and NC-SCs using the db-cAMP free SC differentiation media. After 3 days of culture, the differentiation effect was evaluated by WB analysis, and the results showed that Gnao1-KD-SCs had higher expression of MAG compared with NC-SCs (Fig. 8Bb3), indicating that Gnao1-KD-SCs could differentiate even in the absence of db-cAMP, further demonstrating that deletion of Gnao1 in SCs promotes the formation of cAMP, which compensated for the removal of db-cAMP.

**Fig. 9:**
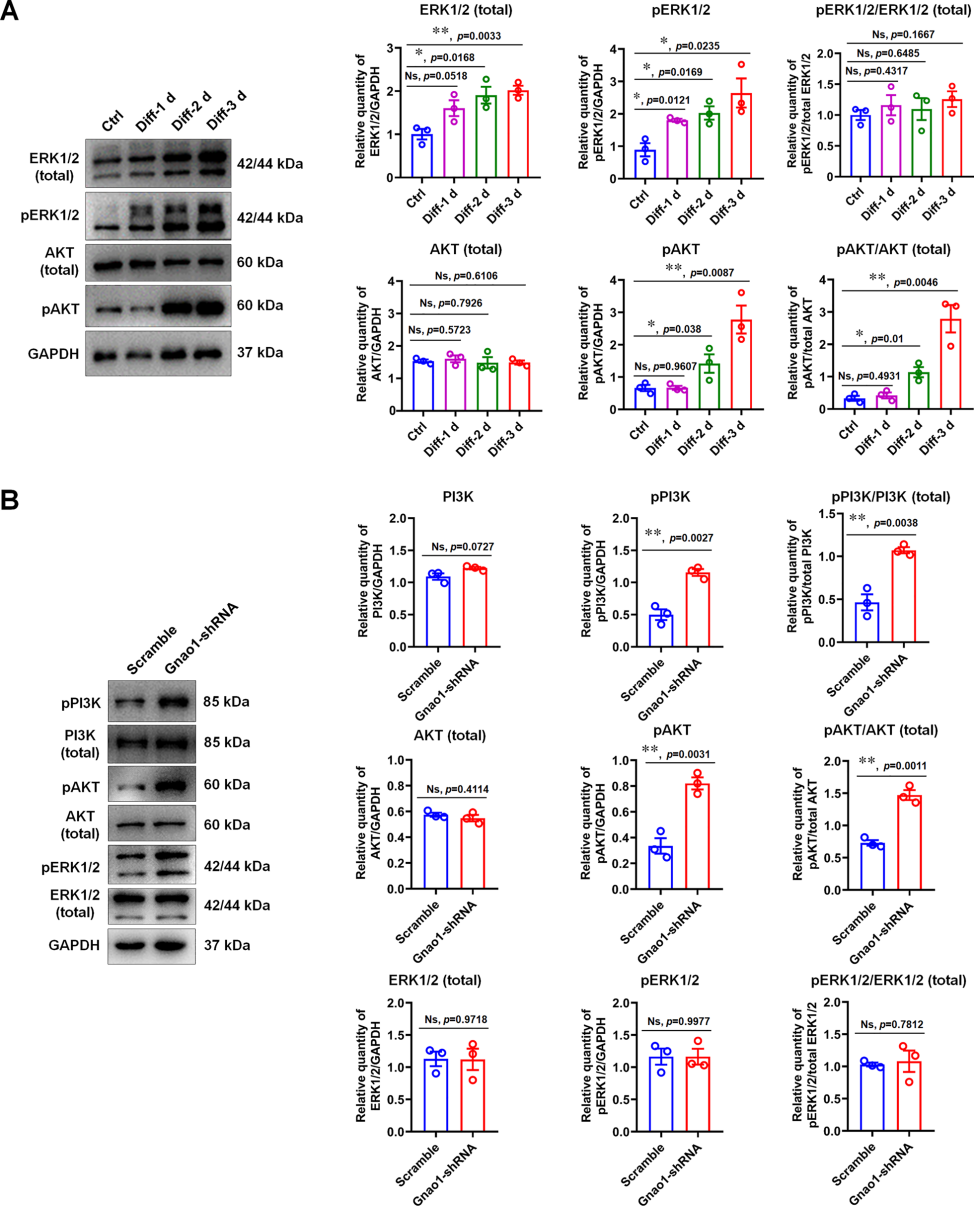
Gnao1 deficiency in SCs result in the activation of PI3K-AKT cascade, contributing to SC differentiation. **A** WB analysis of the activation of PI3K/AKT and MAPK/ERK in the in vitro SC differentiation model, also shown are representative WB images (left) and quantitative statistics (right). *T*-test, n = 3, Ns, *p* > 0.05 vs control, no statistical difference. *, *p* < 0.05 and **, *p* < 0.01 vs control. **B** WB analysis of the activation of PI3K/AKT and MAPK/ERK in differentiated Gnao1-KD-SCs (Gnao1-shRNA) and control SCs (Scramble), also shown are representative WB images (left) and quantitative statistics (right). *T*-test, n = 3, Ns, *p* > 0.05 vs control, no statistical difference. **, *p* < 0.01 vs Scramble

GPCRs binding to heterotrimeric G protein (Gαβγ) promote the recruitment of GTP to the Gα subunit, leading to the release of the Gβγ from Gαβγ trimer, and both free Gα and Gβγ activate their respective downstream signals to affect various biological functions [36]. PI3K/AKT and MAPK/ERK are major downstream signaling pathways of the GPCRs and have been shown to be involved in glial differentiation and myelination in the nervous system [18, 19, 46]. Therefore, we hypothesized that the decreased expression of Gnao1 in SCs would result in a reduction of Gαo proteins encoded by Gnao1, leading to the de-polymerization of Gαβγ and then increase the number of free Gβγ dimers, thereby causing the activation of downstream signaling pathways, such as PI3K/AKT and MAPK/ERK, which would be beneficial to the differentiation of SCs. To prove this, we detected the activation of PI3K/AKT and MAPK/ERK in the in vitro SC differentiation model with WB analysis, and found that, during SC differentiation, the levels of both phosphorylation of ERK1/2 (pERK1/2) and total ERK1/2 (tERK1/2) increased, but no significant difference in the ratio between the two. Unlike MAPK/ERK, the ratio of phosphorylated AKT (pAKT) to total AKT (tAKT) and phosphorylated PI3K (pPI3k) to total PI3K (tPI3k) was significantly different (Fig. 9A), indicating that db-cAMP induced SC differentiation mainly by activating PI3K/AKT signaling pathway. Next, we examined the activation of PI3K/AKT and MAPK/ERK in Gnao1-KD-SCs and NC-SCs to observe the effects of Gnao1 knockdown on the two pathways. As demonstrated by WB, the low expression of Gnao1 in SCs indeed induced higher phosphorylation of PI3K and AKT without affecting ERK1/2 compared to NC-SCs (Fig. 9B), confirming the hypothesis above.

**Fig. 10:**
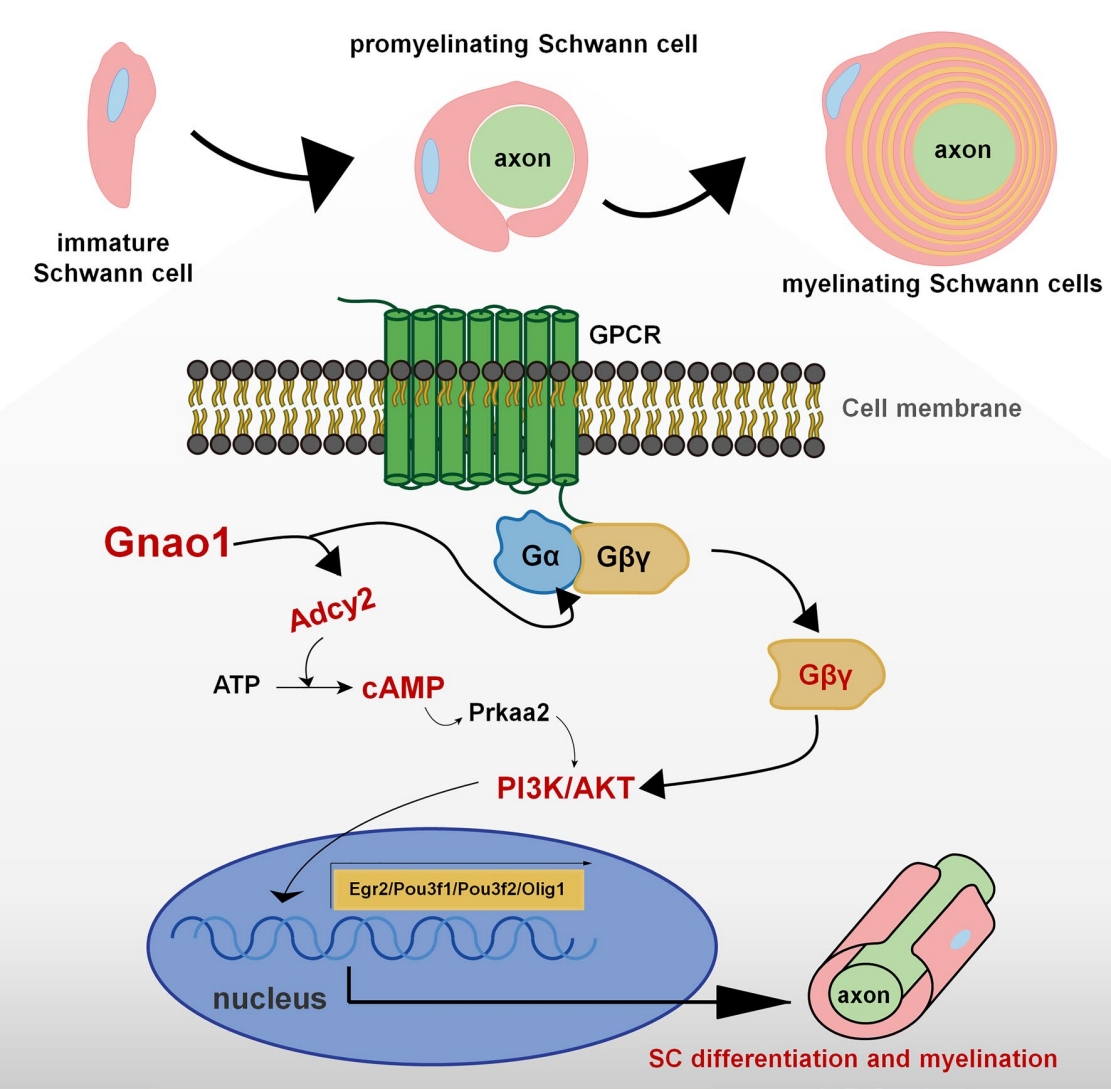
Schematic showing the preliminary mechanism of Gnao1 in SCs regulating SC differentiation and myelination. Gnao1 deficiency triggering spontaneous differentiation of SCs through the following two ways: (1) The absence of Gnao1 in SCs reduced the content of the Gαo, resulting in the depolymerization of Gαβγ heterotrimers, which increased the number of free Gβγ dimers, promoted the activation of downstream signaling pathways (such as PI3K/AKT), and facilitates the differentiation of SCs. (2) Gnao1 deletion in SCs led to increased synthesis of cAMP, the second messenger in the G protein-coupled receptor signaling pathway, which accelerated SCs differentiation

Taken together, we concluded that the reduced expression of Gnao1 in SCs caused the decrease in the content of Gαo proteins, resulting in the de-polymerization of Gαβγ trimers and an increase in the number of free Gβγ dimers, meanwhile increasing Adcy2 expression to accelerate cAMP formation. Afterwards, they cooperatively activated the PI3K/AKT signaling pathway to promote SC differentiation and subsequent myelination (Fig. 10).

## Discussion

Myelin sheath is the ultrastructural in the nervous system, and its abnormality can lead to a variety of myelin-related neurological disorder such as MS. To explore the key molecules regulating myelination is helpful to the treatment of demyelinating diseases. Many studies have shown that G-protein coupled receptors, such as GPR126, GPR37, and GPR44, play critical roles in myelination [25, 47]. However, the roles of G proteins (Gαβγ), especially Gα subunits, remain unclear. Here, we show a novel role for Gnao1, the gene encodes for Gαo protein, in regulating SC differentiation and subsequent myelination in PNS. We found that Gnao1 deficiency in SCs significantly enhanced myelin regeneration in an in vivo re-myelination model, and revealed that Gnao1 is a negative regulator of SC differentiation and that its inhibitory function is achieved by reducing cAMP level and suppression of PI3K-AKT cascade activation.

One of our findings is that only endogenous Gnao1 alterations in SCs affect re-myelination after PNI, whereas changes of Gnao1 in neurons do not (Figs. 2, 3, 4), which actually surprised us because of some studies about Gnao1 mutations resulting in movement disorders [9, 31]. As we known, the possible etiological bases of movement disorder may be explained by examination of the integrity of the myelin sheath structure. In addition, given that myelination requires cooperation between neurons and myelin-forming cells (SCs in PNS and OLs in CNS), we initially thought that changes in the endogenous Gnao1 of these 2 types of cells must have an effect on myelination, but this is not the case. We assumed that the roles of neuronal Gnao1 in movement disorders may be through additional non-myelination-related-mechanism, which was indeed supported by the following some studies. Goldstein [14] and Mercimek-Mahmutoglu [24] reported that Gnao1-related movement disorder was associated with to the presynaptic role of Gαo (Gnao1-encoded G protein) in regulating the release of neurotransmitters including the dopamine, epinephrine, noradrenaline and 5-hydroxytryptamine, etc.. Gnao1 knockout mice showed a strong developmental delay with multiple neurological abnormalities such as severe defects of motor control, and the neurons from Gnao1 knockout mice showed the inhibition of Ca^2+^ channel currents, suggesting that Gnao1 in neurons also regulated motor function by affecting nerve impulses [48]. Based on these reports, we believed that Gnao1 in neurons and SCs achieved regulation of motor function through different mechanisms, that is, Gnao1 in SCs played a role in motor through influencing the structure and formation of myelin sheaths, while Gnao1 in neurons regulated motor function by affecting the transmission of neurotransmitters and nerve impulses. In this study, although we have no enough experimental evidences that clearly explains the speculations, it was observed that the alteration of Gnao1 in SCs (Gnao1-NKD and Gnao1-NOE mice) indeed affected re-myelination and motor function recovery after sciatic nerve injury (Figs. 2, 3), while Gnao1-SKD-mice exhibited no differences in myelin sheath ultrastructure and movement properties versus controls (Fig. 4), supporting that Gnao1 located in SCs and neurons, regulated movement using different mechanisms as described above.

Myelination in the PNS summarized in four main steps: (1) the proliferation and migration of immature SCs, (2) the recognition of target axons, (3) the differentiation of immature SCs into pro-myelinating SCs and (4) myelinating SCs wrapping axon, myelin formation and compaction [32, 42]. All of these steps are regulated in an orderly manner by multiple factors. In this study, we found that Gnao1 knockdown inhibits SC proliferation and migration, but promotes SC differentiation (Figs. 5, 6, 7), indicating that Gnao1 achieves regulation of PNS myelination by coordinating of SC proliferation, migration and differentiation. To explore the molecular mechanisms by which Gnao1 regulates the differentiation of SCs, we sequenced the transcriptome of Gnao1-KD-SCs and control SCs (NCs) before and after differentiation. Surprisingly, we found that inhibiting Gnao1 expression in SCs increased the expression of myelin constitutive proteins (MPZ, MBP, PMP22 and MAG) and some positive regulatory factors such as Olig1, Pou3f2, and Egr2, even before inducing SCs differentiation (Fig. 8A and Additional file 1: Fig. S6, Additional file 3: Table S2), suggesting that the absence of Gnao1 in SCs may lead to spontaneous differentiation of SCs. So how does it work? As well known, that Gαo encoded by Gnao1 usually binds to GDP and forms a heterotrimer with Gβγ. When stimulated by extracellular signals, the GDP bound by Gαo is exchanged with GTP, causing conformational change of Gαo to dissociate the Gβγ from the heterotrimer [16, 44]. On the one hand, the free Gαo inhibits the activity of adenylate cyclase (AC) from converting adenosine triphosphate (ATP) into an important second messenger, cAMP, thereby suppressing its downstream signaling [36]. On the other hand, free Gβγ also regulate cellular function by activating downstream signaling pathways [9, 31, 38]. Therefore, we speculated that the possible mechanism of Gnao1 deficiency triggering spontaneous differentiation of SCs is as follows: (1) The absence of Gnao1 in SCs reduced the content of the Gαo, resulting in the depolymerization of Gαβγ heterotrimers, which increased the number of free Gβγ dimers, promoted the activation of downstream signaling pathways (such as PI3K/AKT and MAPK/ERK), and facilitates the differentiation of SCs. (2) Gnao1 deletion in SCs led to increased synthesis of cAMP, the second messenger in the G protein-coupled receptor signaling pathway, which accelerated SCs differentiation. In fact, we did notice that the increased expression of Adcy2 and Prkaa2 (protein kinase AMP-activated catalytic subunit alpha 2, Prkaa2) in Gnao1-KD-SCs compared to NCs (Additional file 1: Fig. S6, Additional file 3: Table S2). Adcy2, has been found to convert ATP to cAMP, which activates the protein kinase PKA (Prkaa2), leading to activation of the PI3K-AKT pathway. Furthermore, we also found that the functions of up-regulated DEGs between Gnao1-KD-SCs and NCs were related to cAMP and PI3K/AKT signaling pathway by KEGG analysis (Fig. 8A, Additional file 3: Table S2). We found through WB analysis that compared with NCs, the expression of Adcy2 in Gnao1-KD-SCs was indeed increased, and ELISA results also evidenced the elevated cAMP content in Gnao1-KD-SCs (Fig. 8Bb1-2). Next, we used the SC differentiation medium without db-cAMP to culture Gnao1-KD-SCs, and found that Gnao1-KD-SCs had higher expression of myelin-related protein MAG compared with NCs (Fig. 8Bb3), suggesting that knockdown of Gnao1 expression in SCs could promote the formation of cAMP, which compensates for the effect of removed db-cAMP. Moreover, our data showed that Gnao1 knockdown only affected the PI3K/AKT signaling pathway, which is involved in glial differentiation and myelination of the nervous system [18, 19, 46], but had no effect on MAPK/ERK signaling (Fig. 9). Taken together, we believe that down-regulation of Gnao1 expression in SCs can increase cAMP content and the number of free Gβγ dimer, resulting in activation of PI3K/AKT signaling pathway, promoting differentiation of SCs.

## Conclusion

Gnao1 is important for myelination in PNS. Gnao1 knockdown in SCs promotes the axonal re-myelination and motor function recovery after nerve injury. Conversely, mice with Gnao1 overexpression in SCs display the insufficient myelinating capacity and delayed re-myelination. Gnao1 deletion in SCs promotes SC differentiation by the elevation of cAMP content and the activation of PI3K/AKT pathway (Fig. 10). In light of the current data, our findings uncover a function of Gnao1 to negatively regulate SC differentiation, identifying a novel candidate drug target for the treatment of demyelinating diseases.

### Supplementary Information

**Additional file 1**: **Fig. S1.** Gnao1 expression in nerve tissue (sciatic nerves and spinal cords) and myelination-related cells (neurons and SCs) by RT-qPCR (**A**) and WB analysis (**B**). **Fig. S2** Validation of Gnao1-shRNAs interference efficiency. **A** RT-qPCR comparing the mRNA levels of Gnao1 in SCs treated with Gnao1-shRNAs or Scramble (NC-shRNA) for 48 h. *T*-test, n = 3, **, *p* < 0.01 and ***, *p* < 0.001 vs NC-shRNA. **B** WB comparing the protein levels of Gnao1 in SCs treated with Gnao1-shRNAs or Scramble for 48 h. *T*-test, n = 3, *, *p* < 0.05 and **,* p* < 0.01 vs NC-shRNA. **Fig. S3** Knockdown or overexpression of Gnao1 level in Schwann cells of mouse sciatic nerve by injection of virus carried with Gnao1-shRNA or mouse Gnao1 coding sequence, respectively. **A** Schematic diagram illustrates the experimental process, that is, by respectively injecting the viruses carrying the Gnao1-shRNA or coding sequences into sciatic nerve, to generate mice with Gnao1 knockdown or overexpression in the sciatic nerve (referred to as Gnao1-NKD and Gnao1-NOE-mice). **B** and **C** WB comparing the protein levels of Gnao1 in Gnao1-NKD-mice (**B**), Gnao1-NOE-mice (**C**) together with their own controls, showing the lower Gnao1 expression in Gnao1-NKD-mice and higher Gnao1 expression in Gnao1-NOE-mice compared to controls, and IHC showing the Gnao1 knockdown or overexpression occurred mainly in the SCs of the sciatic nerves. Scale bar = 100 μm. *T*-test, n = 3, **, *p* < 0.01 and ****, *p* < 0.0001 vs controls. **Fig. S4** Knockdown Gnao1 expression in neurons of mouse spinal cord by injection of virus carried with Gnao1-shRNA. **A** Schematic diagram illustrates the experimental process, that is, by intrathecally injecting the viruses carrying the Gnao1-shRNA into spinal cord, to generate mice with Gnao1 knockdown in the spinal cord (referred to as Gnao1-SKD-mice). **B** WB comparing the protein levels of Gnao1 in Gnao1-SKD-mice together with controls, showing the lower Gnao1 expression in Gnao1-SKD-mice compared to controls. *T*-test, n = 3, **, *p* < 0.01 vs controls.** C** IHC showing the Gnao1 knockdown occurred mainly in the spinal cord neurons. Scale bar = 500 μm. Zoomed in is the enlargement of the white box area, scale bar = 200 μm. **Fig. S5.** Analysis of RNA sequencing data of Gnao1-KD-SCs and control (NC) before and after differentiation. **A** Bar chart showing the number of differentially expressed genes (DEGs) obtained by pair-to-pair comparison of NC-SCs, Gnao1-KD-SCs, Diff-NC-SCs and Diff-Gnao1-KD-SCs (cutoff: fold change (FC) > 1.5 or < 0.67 plus *p*-value < 0.05). **Bb1-Ee1** Heat map showing representative DEGs in 4 datasets (i.e., 1960 DEGs in Gnao1-KD-SCs versus NCs (**b1**), 3607 DEGs in Diff-NC-SCs versus NCs (**c1**), 4672 DEGs in Diff-Gnao1-KD-SCs versus Gnao1-KD-SCs (**d1**), and 161 DEGs in Diff-Gnao1-KD-SCs versus Diff-NC-SCs (**e1**)) by cluster heatmap analysis. **Bb2-Ee2** Bubble map showing the top 20 functions of upregulated DEGs in 4 datasets (i.e., Gnao1-KD-SCs versus NCs (**b2**), Diff-NC-SCs versus NCs (**c2**), Diff-Gnao1-KD-SCs versus Gnao1-KD-SCs (**d2**), and Diff-Gnao1-KD-SCs versus Diff-NC-SCs (**e2**)) by KEGG enrichment analysis. **Fig. S6.** Analyze of myelination-related genes in RNA sequencing data with heat map cluster and qPCR. **A** Heat map showing representative myelination-related DEGs in 4 datasets (i.e., DEGs in Gnao1-KD-SCs versus NCs, Diff-NC-SCs versus NCs, Diff-Gnao1-KD-SCs versus Gnao1-KD-SCs, and Diff-Gnao1-KD-SCs versus Diff-NC-SCs) by cluster heatmap analysis. **B** Histogram showing the relative expression of (**b1**) myelin-forming proteins (MPZ, MBP, PMP22 and MAG), (**b2**) positive transcriptional regulators of myelination (EGR2, Pou3F1, Pou3F2 and Id2), (**b3**) the molecules that have been proven to affect myelination (Dok4, Vamp7, Erbb2, Erbb3, Cadm3 and Adcy2), and others (Gnao1 and Adcy2) in NC-SCs, Gnao1-KD-SCs, Diff-NC-SCs and Diff-Gnao1-KD-SCs. Pair *t*-test, n = 3, Ns, *p* > 0.05, no statistical difference. *, *p* < 0.05, **, *p* < 0.01, and ***, *p* < 0.001

**Additional file 2**: **Table S1.** The analysis of microarray data (GSE163132).

**Additional file 3**: **Table S2.** The analysis of RNA-seq data of Gnao1-KD-SCs and control (NC) before and after differentiation.

## Data Availability

Microarray expression data for SCs at various stages of myelination (i.e., immature, pre-myelination, and myelination) was supplied in NCBI Gene Expression Omnibus (Accession number: GSE163132). The other datasets that support the findings of the current study are available from the corresponding author upon reasonable request.

## Abbreviations

NDDs: Neurodegenerative diseases
PNS: Peripheral nervous system
SCs: Schwann cells
DRG: Dorsal root ganglion
Gnao1-NKD mice: Mice with Gnao1 knockdown in the sciatic nerve
Gnao1-NOE mice: Mice with Gnao1 overexpression in the sciatic nerve
Gnao1-SKD mice: Mice with Gnao1 knockdown in spinal cords
db-cAMP: Dibutyryl cyclic AMP
Ara-C: Cytosine arabinoside
PDL: Poly-D-lysine
Nrg1: Neuregulin 1
EdU: 5-Ethynyl-2'-deoxyuridine
AAV: Adeno-associated virus
IHC: Immunohistochemistry
ICC: Immunocytochemistry
RT-qPCR: Real-time quantitative PCR
WB: Western blotting
TEM: Transmission electron microscopy
GPR: G-protein coupled receptors
MAG: Myelin associated glycoprotein
P0 (MPZ): Myelin protein zero
MBP: Myelin basic protein
PMP22: Peripheral myelin protein-22
PI3K: Phosphatidylinositol 3-kinase
TuJ1: β-III tubulin
RNA-seq: RNA sequencing
GO: Gene ontology
KEGG: Kyoto encyclopedia of genes and genomes
