# Supplementary material for: The Schwann cell-specific G-protein Gαo (Gnao1) is a cell-intrinsic controller contributing to the regulation of myelination in peripheral nerve system
Source: Acta Neuropathol Commun. 2024 Feb 8;12:24. doi: 10.1186/s40478-024-01720-3 (PMC10854112; doi:10.1186/s40478-024-01720-3)
Supplement: Supplementary file 1 — Additional file 1: Fig. S1. Gnao1 expression in nerve tissue (sciatic nerves and spinal cords) and myelination-related cells (neurons and SCs) by RT-qPCR (A) and WB analysis (B). Fig. S2 Validation of Gnao1-shRNAs interference efficiency. A RT-qPCR comparing the mRNA levels of Gnao1 in SCs treated with Gnao1-shRNAs or Scramble (NC-shRNA) for 48 h. T-test, n = 3, **, p < 0.01 and ***, p < 0.001 vs NC-shRNA. B WB comparing the protein levels of Gnao1 in SCs treated with Gnao1-shRNAs or Scramble for 48 h. T-test, n = 3, *, p < 0.05 and **, p < 0.01 vs NC-shRNA. Fig. S3 Knockdown or overexpression of Gnao1 level in Schwann cells of mouse sciatic nerve by injection of virus carried with Gnao1-shRNA or mouse Gnao1 coding sequence, respectively. A Schematic diagram illustrates the experimental process, that is, by respectively injecting the viruses carrying the Gnao1-shRNA or coding sequences into sciatic nerve, to generate mice with Gnao1 knockdown or overexpression in the sciatic nerve (referred to as Gnao1-NKD and Gnao1-NOE-mice). B and C WB comparing the protein levels of Gnao1 in Gnao1-NKD-mice (B), Gnao1-NOE-mice (C) together with their own controls, showing the lower Gnao1 expression in Gnao1-NKD-mice and higher Gnao1 expression in Gnao1-NOE-mice compared to controls, and IHC showing the Gnao1 knockdown or overexpression occurred mainly in the SCs of the sciatic nerves. Scale bar = 100 μm. T-test, n = 3, **, p < 0.01 and ****, p < 0.0001 vs controls. Fig. S4 Knockdown Gnao1 expression in neurons of mouse spinal cord by injection of virus carried with Gnao1-shRNA. A Schematic diagram illustrates the experimental process, that is, by intrathecally injecting the viruses carrying the Gnao1-shRNA into spinal cord, to generate mice with Gnao1 knockdown in the spinal cord (referred to as Gnao1-SKD-mice). B WB comparing the protein levels of Gnao1 in Gnao1-SKD-mice together with controls, showing the lower Gnao1 expression in Gnao1-SKD-mice compared to controls. T-test, n = [file 40478_2024_1720_MOESM1_ESM.docx]

**Supplementary Figures and figure legends:**


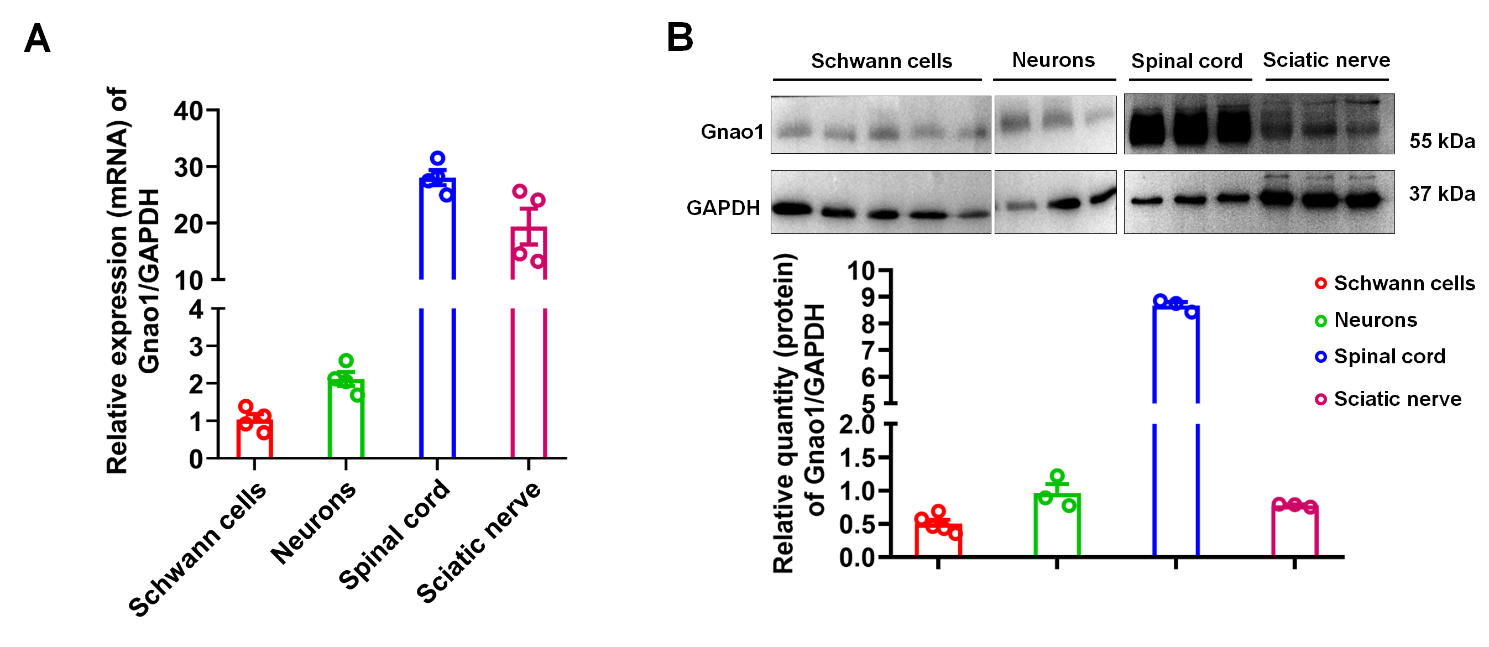


**Fig.S1** Gnao1 expression in nerve tissue (sciatic nerves and spinal cords) and myelination-related cells (neurons and SCs) by RT-qPCR (**A**) and WB analysis (**B**)


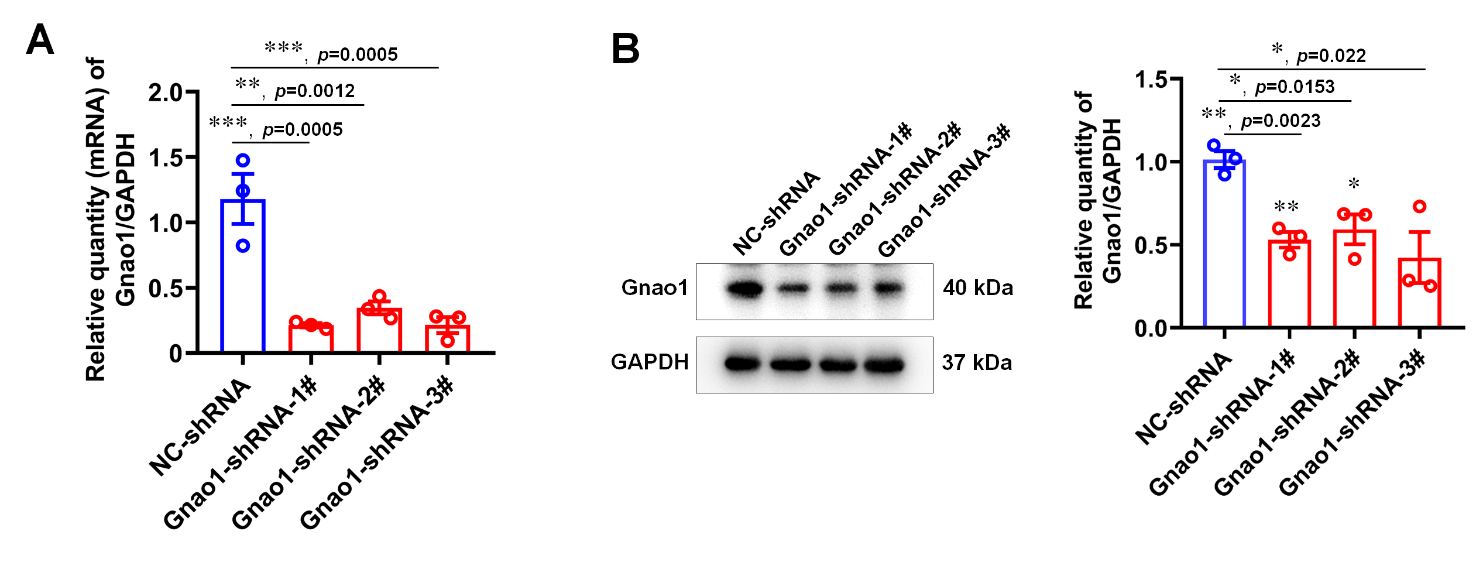


**Fig. S2** Validation of Gnao1-shRNAs interference efficiency. **A** RT-qPCR comparing the mRNA levels of Gnao1 in SCs treated with Gnao1-shRNAs or Scramble (NC-shRNA) for 48 hours. *T*-test, n=3, **, *p* < 0.01 and ***, *p* < 0.001 vs NC-shRNA. **B** WB comparing the protein levels of Gnao1 in SCs treated with Gnao1-shRNAs or Scramble for 48 hours. *T*-test, n=3, *, *p* < 0.05 and **, *p* < 0.01 vs NC-shRNA.


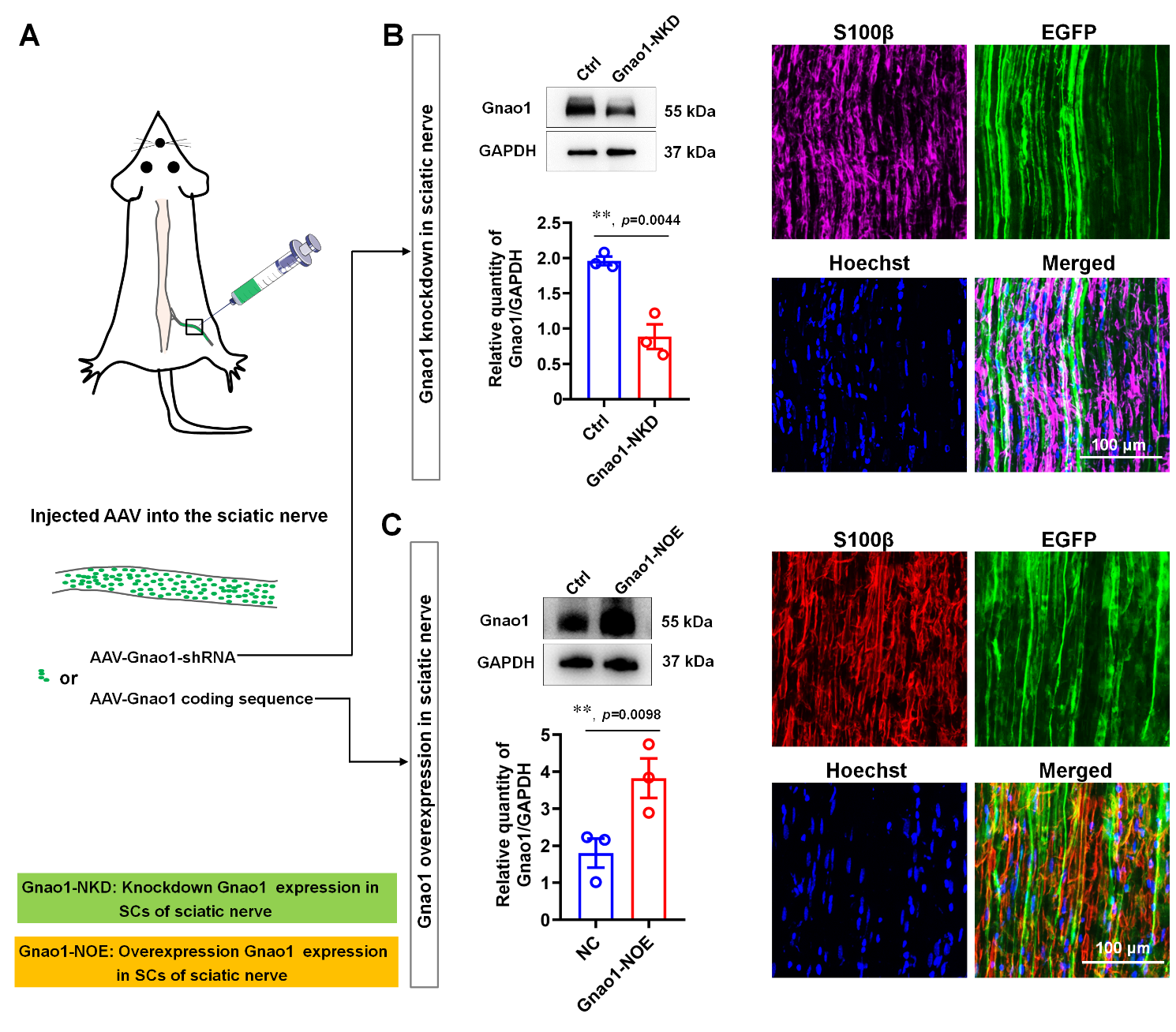


**Fig. S3.** Knockdown or overexpression of Gnao1 level in Schwann cells of mouse sciatic nerve by injection of virus carried with Gnao1-shRNA or mouse Gnao1 coding sequence, respectively. **A** Schematic diagram illustrates the experimental process, that is, by respectively injecting the viruses carrying the Gnao1-shRNA or coding sequences into sciatic nerve, to generate mice with Gnao1 knockdown or overexpression in the sciatic nerve (referred to as Gnao1-NKD and Gnao1-NOE-mice). **B** and **C** WB comparing the protein levels of Gnao1 in Gnao1-NKD-mice (**B**), Gnao1-NOE-mice (**C**) together with their own controls, showing the lower Gnao1 expression in Gnao1-NKD-mice and higher Gnao1 expression in Gnao1-NOE-mice compared to controls, and IHC showing the Gnao1 knockdown or overexpression occurred mainly in the SCs of the sciatic nerves. Scale bar = 100 μm. *T*-test, n=3, **, *p* < 0.01 and ****, *p* < 0.0001 vs controls.


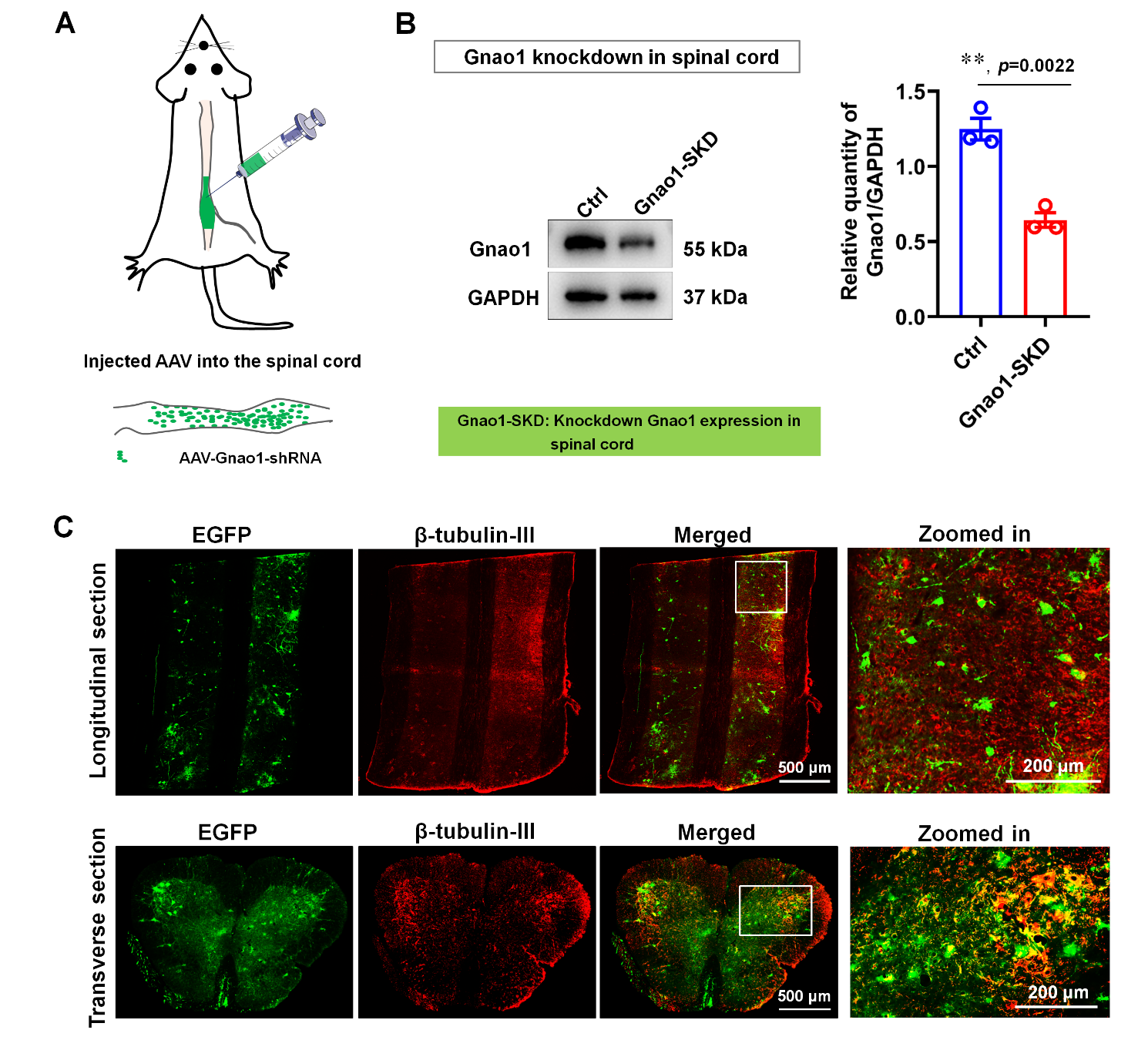


**Fig. S4.** Knockdown Gnao1 expression in neurons of mouse spinal cord by injection of virus carried with Gnao1-shRNA. **A** Schematic diagram illustrates the experimental process, that is, by intrathecally injecting the viruses carrying the Gnao1-shRNA into spinal cord, to generate mice with Gnao1 knockdown in the spinal cord (referred to as Gnao1-SKD-mice). **B** WB comparing the protein levels of Gnao1 in Gnao1-SKD-mice together with controls, showing the lower Gnao1 expression in Gnao1-SKD-mice compared to controls. *T*-test, n=3, **, *p* < 0.01 vs controls. **C** IHC showing the Gnao1 knockdown occurred mainly in the spinal cord neurons. Scale bar = 500 μm. Zoomed in is the enlargement of the white box area, scale bar = 200 μm


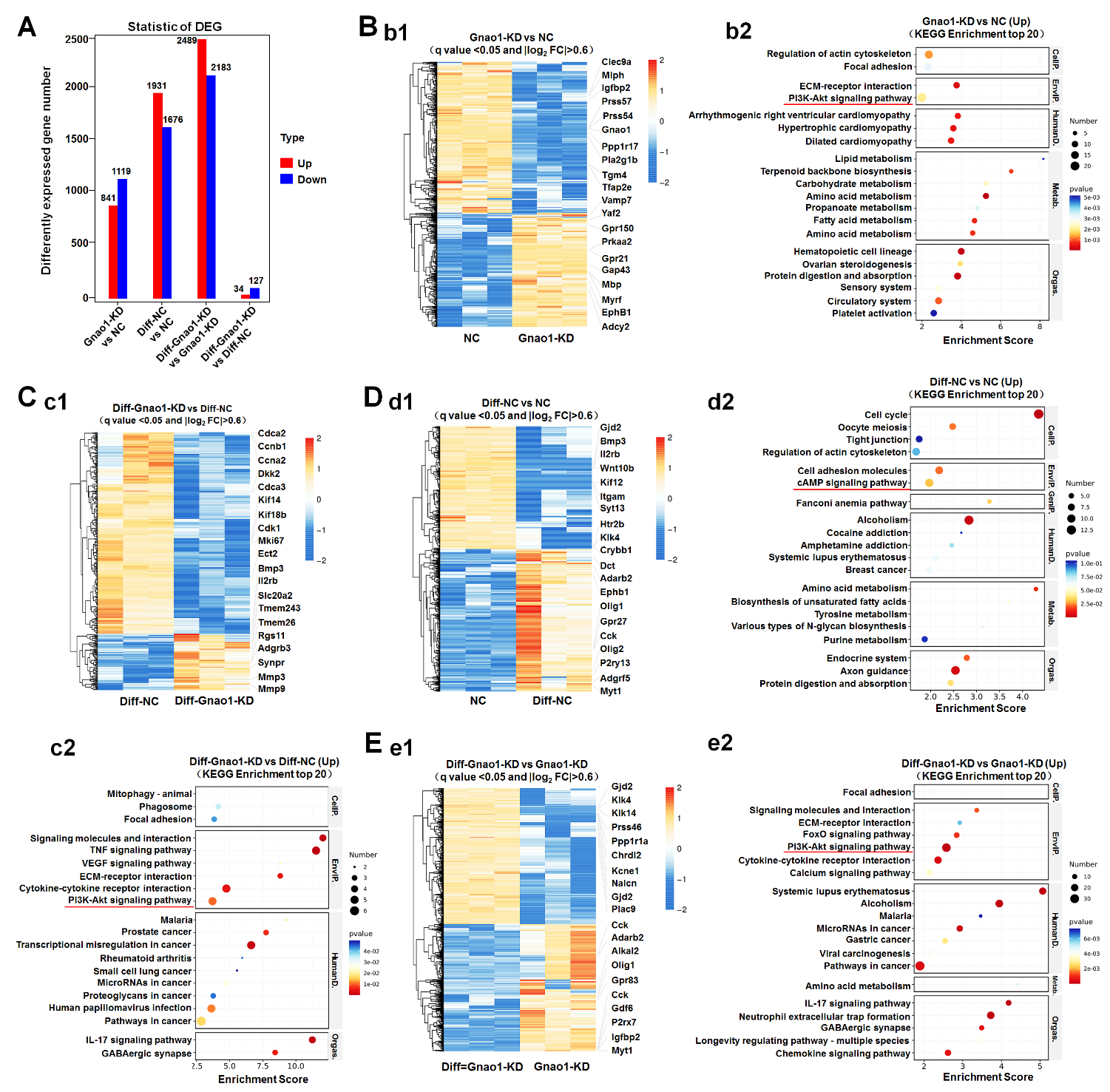


**Fig. S5.** Analysis of RNA sequencing data of Gnao1-KD-SCs and control (NC) before and after differentiation. **A** Bar chart showing the number of differentially expressed genes (DEGs) obtained by pair-to-pair comparison of NC-SCs, Gnao1-KD-SCs, Diff-NC-SCs and Diff-Gnao1-KD-SCs (cutoff: fold change (FC) > 1.5 or < 0.67 plus *p*-value < 0.05). **Bb1-Ee1** Heat map showing representative DEGs in 4 datasets (i.e., 1960 DEGs in Gnao1-KD-SCs versus NCs (**b1**), 3607 DEGs in Diff-NC-SCs versus NCs (**c1**), 4672 DEGs in Diff-Gnao1-KD-SCs versus Gnao1-KD-SCs (**d1**), and 161 DEGs in Diff-Gnao1-KD-SCs versus Diff-NC-SCs (**e1**)) by cluster heatmap analysis. **Bb2-Ee2** Bubble map showing the top 20 functions of upregulated DEGs in 4 datasets (i.e., Gnao1-KD-SCs versus NCs (**b2**), Diff-NC-SCs versus NCs (**c2**), Diff-Gnao1-KD-SCs versus Gnao1-KD-SCs (**d2**), and Diff-Gnao1-KD-SCs versus Diff-NC-SCs (**e2**)) by KEGG enrichment analysis

**
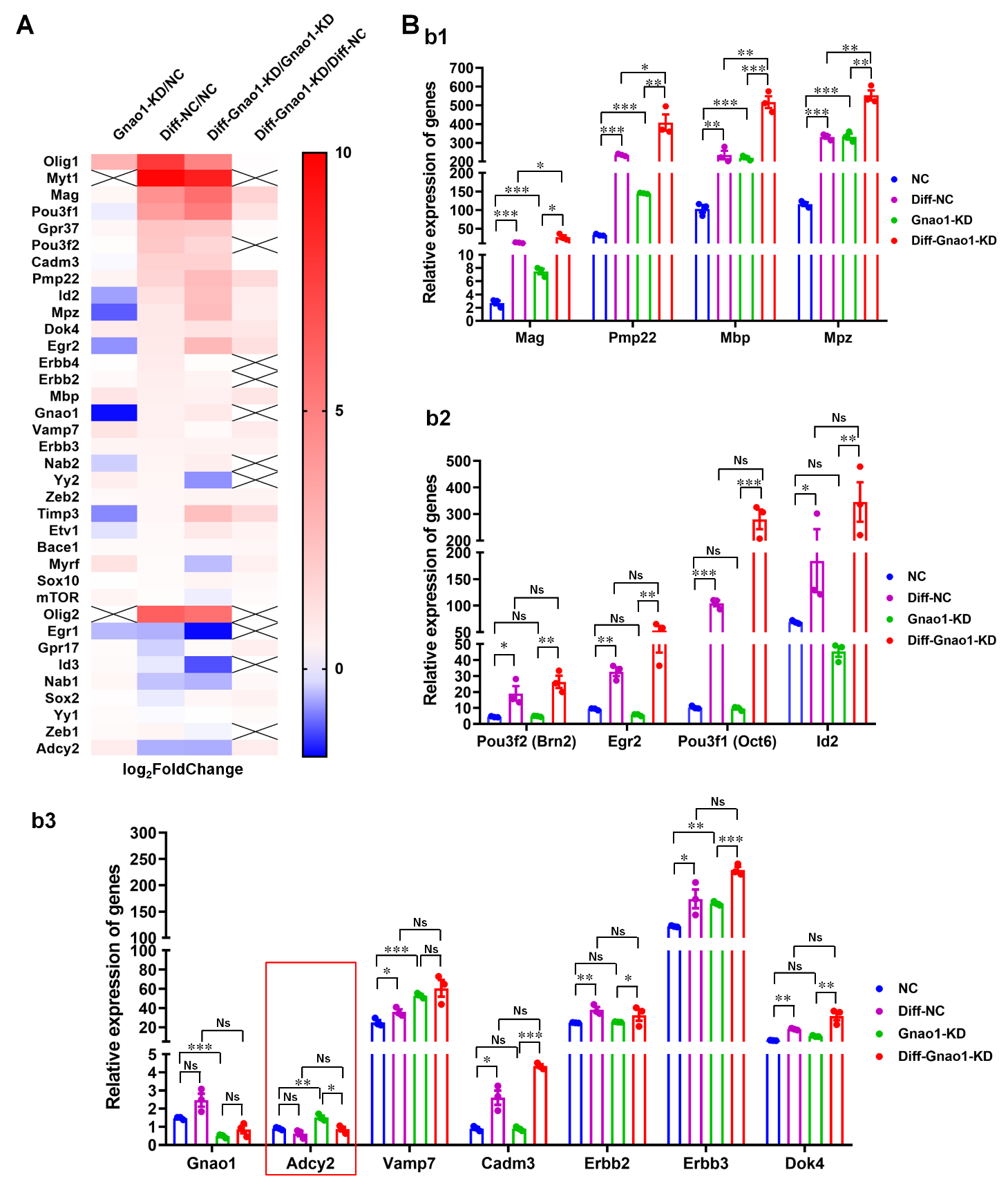
**

**Fig. S6.** Analyze of myelination-related genes in RNA sequencing data with heat map cluster and qPCR. **A** Heat map showing representative myelination-related DEGs in 4 datasets (i.e., DEGs in Gnao1-KD-SCs versus NCs, Diff-NC-SCs versus NCs, Diff-Gnao1-KD-SCs versus Gnao1-KD-SCs, and Diff-Gnao1-KD-SCs versus Diff-NC-SCs) by cluster heatmap analysis. **B** Histogram showing the relative expression of (**b1**) myelin-forming proteins (MPZ, MBP, PMP22 and MAG), (**b2**) positive transcriptional regulators of myelination (EGR2, Pou3F1, Pou3F2 and Id2), (**b3**) the molecules that have been proven to affect myelination (Dok4, Vamp7, Erbb2, Erbb3, Cadm3 and Adcy2), and others (Gnao1 and Adcy2) in NC-SCs, Gnao1-KD-SCs, Diff-NC-SCs and Diff-Gnao1-KD-SCs. Pair *t*-test, n=3, Ns, *p* > 0.05, no statistical difference. *, *p* < 0.05, **, *p* < 0.01, and ***, *p*<0.001.
